# Superior Anticancer and Antifungal Activities of New Sulfanyl-Substituted Niclosamide Derivatives

**DOI:** 10.3390/biomedicines12071621

**Published:** 2024-07-21

**Authors:** Jingyi Ma, Dileepkumar Veeragoni, Hindole Ghosh, Nicole Mutter, Gisele Barbosa, Lauren Webster, Rainer Schobert, Wendy van de Sande, Prasad Dandawate, Bernhard Biersack

**Affiliations:** 1 Department of Medical Microbiology and Infectious Diseases, Erasmus MC, University Medical Center Rotterdam, Dr. Molewaterplein 40, 3015 GD Rotterdam, The Netherlands; m.jingyi@erasmusmc.nl; 2 Department of Cancer Biology, University of Kansas Medical Center, 3901 Rainbow Boulevard, Kansas City, KS 66160, USA; dveeragoni@kumc.edu (D.V.); hghosh@kumc.edu (H.G.); 3 Wellcome Centre—Antiinfectives Research, School of Life Sciences, University of Dundee, Nethergate, Dundee DD1 4HN, UK; n.l.mutter@dundee.ac.uk (N.M.); bgiselejf@gmail.com (G.B.); l.a.webster@dundee.ac.uk (L.W.); 4 Organic Chemistry Laboratory, University Bayreuth, Universitätsstrasse 30, 95440 Bayreuth, Germany; rainer.schobert@uni-bayreuth.de

**Keywords:** niclosamide, salicylanilide, fluorine, anticancer agents, antifungal agents, esophageal carcinoma, eumycetoma, STAT3, β-catenin, *Madurella mycetomatis*

## Abstract

The approved anthelmintic salicylanilide drug niclosamide has shown promising anticancer and antimicrobial activities. In this study, new niclosamide derivatives with trifluoromethyl, trifluoromethylsulfanyl, and pentafluorosulfanyl substituents replacing the nitro group of niclosamide were prepared (including the ethanolamine salts of two promising salicylanilides) and tested for their anticancer activities against esophageal adenocarcinoma (EAC) cells. In addition, antifungal activity against a panel of *Madurella mycetomatis* strains, the most abundant causative agent of the neglected tropical disease eumycetoma, was evaluated. The new compounds revealed higher activities against EAC and fungal cells than the parent compound niclosamide. The ethanolamine salt **3a** was the most active compound against EAC cells (IC_50_ = 0.8–1.0 µM), and its anticancer effects were mediated by the downregulation of anti-apoptotic proteins (BCL2 and MCL1) and by decreasing levels of β-catenin and the phosphorylation of STAT3. The plausibility of binding to the latter factors was confirmed by molecular docking. The compounds **2a** and **2b** showed high in vitro antifungal activity against *M. mycetomatis* (IC_50_ = 0.2–0.3 µM) and were not toxic to *Galleria mellonella* larvae. Slight improvements in the survival rate of *G. mellonella* larvae infected with *M. mycetomatis* were observed. Thus, salicylanilides such as **2a** and **3a** can become new anticancer and antifungal drugs.

## 1. Introduction

Salicylic acid and its prominent natural and synthetic derivatives possess considerable biological activities [1]. Niclosamide is a salient salicylanilide-based antiparasitic drug developed by Bayer which has been widely applied for the therapy of tapeworm infections since the 1960s [2]. Repurposing studies of this drug revealed promising anticancer and antimicrobial activities [3,4]. The anticancer effects of niclosamide were associated with its function as a mitochondrial uncoupler and its ability to form toxic reactive oxygen and nitrogen species (RONS). Interference with vital cellular signaling pathways (e.g., Wnt/β-catenin, Notch, STAT3, mTOR, or NF-κB) was also documented for niclosamide and structurally related salicylanilides in various cancers [5,6]. 

Esophageal cancer (EC) is characterized by high global annual incidences (604,100 new cases) and mortality rates (544,076 deaths) with a rising tendency (an increase of 35–37% until 2030). Hot spots for EC are in East, Central, and South Asia, South and East Africa, and Northwest Europe [7,8]. Thus, EC is a growing health problem since a late diagnosis of rapidly growing EC is the common case leading to a poor prognosis with 5-year survival rates below 20% [8,9,10]. The aggressive clinical features render EC a highly challenging cancer disease [11,12]. There are two basic EC subtypes: esophageal squamous cell carcinoma (ESCC) and esophageal adenocarcinoma (EAC). While ESCC is predominant globally, EAC incidence is rising especially in Western countries (two-thirds of EC cases) due to obesity and associated diseases such as gastroesophageal reflux disease and Barrett’s esophagus [13,14]. 

Therapy of EC is confined to endoscopy, surgery, and platinum-based chemoradiotherapy [15,16]. Niclosamide has also been studied in EC models and showed promising results in paclitaxel-resistant EC by the suppression of Wnt/β-catenin [17]. In addition, niclosamide suppressed STAT3 (signal transducer and activator of transcription 3) signaling in EC cells and sensitized them to commonly applied anticancer drugs such as cisplatin and paclitaxel [18]. Of note, increased mitochondrial respiration and glycolytic activity were associated with an enhanced aggressive EAC phenotype, while mitochondrial uncoupling has generally become a suitable target to overcome cancer resistance [19,20,21].

**Figure 1:**
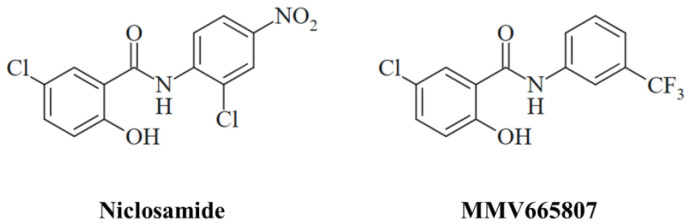
The structures of the antifungal salicylanilides niclosamide and MMV665807.

The antimicrobial activities of niclosamide are broad in scope following the paradigm of a “magic blanket”, and the drug exerted especially promising results as an antiviral agent [22,23]. The antifungal properties of niclosamide and related salicylanilides are likewise sound and diverse affecting several human pathogenic fungi including *Candida* spp., *Trichophyton* spp., *Cryptococcus neoformans*, *Sporothrix brasiliensis*, and *Madurella mycetomatis* [24]. The latter pathogen, *M. mycetomatis*, is the causative agent of fungal mycetoma (eumycetoma), which is endemic in the vast regions of the “mycetoma belt” in Asia and Africa and which is classified as a neglected tropical disease (NTD) since current treatment options are inefficient and/or unavailable for the patients [25]. Surgery and the antifungal itraconazole are currently applied for the treatment of eumycetoma [26]. Fosravuconazole displayed promising results in a recent clinical trial with mycetoma patients and might replace itraconazole in the future [27]. Niclosamide, its ethanolamine salt, and the trifluoromethyl substituted salicylanilide derivative MMV665807 showed high activities against two East African *M. mycetomatis* isolates (Figure 1) [28]. Notably, niclosamide was also active against *S. brasiliensis*, a fungal pathogen responsible for the formation of sporotrichosis which is another fungal NTD endemic in Brazil [29]. 

Niclosamide has some drawbacks such as genotoxicity based on its chemical structure. It is noteworthy that niclosamide analogs lacking the mutagenic nitro-substituent retained biological activity, and trifluoromethyl substituents (CF_3_) appear to be especially promising in this regard [28,30,31,32]. The CF_3_ group is widely applied in drug design because of its substantial pharmacological properties and can be found in several FDA-approved drugs now [33]. Variations such as trifluoromethoxy (OCF_3_) and trifluoromethylsulfanyl (SCF_3_) substituents revealed pronounced antigiardial and antibiotic activities [34]. The pentafluorosulfanyl (SF_5_) group is known as the “super trifluoromethyl group” and was successfully applied for the synthesis of an improved antimalarial 8-SF_5_ mefloquine analog with higher in vivo activity and a longer half-life than the parent compound [35,36]. In addition, curcuminoids with SF_5_ substituents showed distinctly increased anticancer activities [37,38]. In the present report, we disclose the synthesis of new CF_3_- and SF_5_-substituted niclosamide derivatives and the ethanolamine salts of the two most promising analogs together with their activities against EAC and *M. mycetomatis*.

## 2. Materials and Methods

### 2.1. General Procedures

Column chromatography: silica gel 60 (230–400 mesh, Merck, Darmstadt, Germany). Melting points (uncorrected), Electrothermal 9100 (Thermo Fisher Scientific, Geel, Belgium); IR spectra, Perkin Elmer Spectrum One FT-IR spectrophotometer with ATR sampling unit (Perkin Elmer, Rodgau, Germany; NMR spectra, Bruker Avance 300 spectrometer (Bruker, Billerica, MA, USA); chemical shifts are given in parts per million (*δ*) downfield from tetramethylsilane as the internal standard; mass spectra, UPLC/Orbitrap (ESI-HRMS, Thermo Fisher Scientific, Geel, Belgium). 

### 2.2. Materials

Starting compounds and reagents were obtained from abcr (Karlsruhe, Germany), Alfa Aesar (Karlsruhe, Germany), Sigma-Aldrich (Darmstadt, Germany), and TCI (Zwijndrecht, Belgium). Niclosamide was purchased from Cayman Chemical (Ann Arbor, MI, USA) for anticancer tests or taken from the COVID-19 response box of MMV/Medicines for Malaria Venture (compound MMV003461) for antifungal tests. The known compounds **2d** and **2e** were prepared and analyzed according to the literature [39,40].

### 2.3. Synthesis

#### 2.3.1. Synthesis of New Niclosamide Derivatives—Typical Procedure

Around 0.345 g of 5-chloro-2-hydroxybenzoic acid (2.0 mmol), substituted aniline (1.0 mmol), and 0.385 g of EDCI × HCl (2.0 mmol) were dissolved in CH_2_Cl_2_ (10 mL), and the reaction mixture was stirred at room temperature for 24 h. The solution was concentrated under reduced pressure and purified by column chromatography (silica gel 60).

**5-Chloro-2-hydroxy-*N*-(4-pentafluorosulfanylphenyl)benzamide (2a)**

Yield: 122 mg (0.33 mmol, 33%); colorless solid of m.p. 222–223 °C; *R*_f_ = 0.47 (ethyl acetate/*n*-hexane, 1:3); *υ*_max_(ATR)/cm^−1^ 3006, 1614, 1558, 1506, 1481, 1418, 1350, 1334, 1286, 1221, 1192, 1105, 871, 849, 839, 825, 814, 711, 674, 655; ^1^H NMR (300 MHz, acetone-d_6_) *δ* 7.03 (1 H, d, J = 8.9 Hz), 7.48 (1 H, d, J = 8.9 Hz), 7.8–8.0 (2 H, m), 8.0–8.1 (3 H, m), 10.21 (1 H, s), 11.7–11.8 (1 H, br s); ^13^C NMR (75.5 MHz, acetone-d_6_) *δ* 117.7, 120.6, 121.6, 124.4, 127.7, 128.5, 130.2, 130.6, 135.3, 142.1, 149.8–150.3 (m), 160.3, 168.2; HRMS for C_13_H_10_O_2_NClF_5_S [M^+^ + H] calcd. 374.00354, found 374.00250.

**5-Chloro-2-hydroxy-*N*-(4-trifluoromethylsulfanylphenyl)benzamide (2b)**

Yield: 172 mg (0.50 mmol, 50%); colorless solid of m.p. 200–201 °C; *R*_f_ = 0.56 (ethyl acetate/*n*-hexane, 1:3); *υ*_max_(ATR)/cm^−1^ 3322, 3073, 2943, 2777, 2719, 2568, 1628, 1605, 1589, 1537, 1495, 1420, 1398, 1364, 1322, 1292, 1245, 1220, 1175, 1157, 1142, 1107, 1084, 1013, 968, 894, 855, 827, 818, 773, 756, 724, 703, 683, 654; ^1^H NMR (300 MHz, acetone-d_6_) *δ* 7.04 (1 H, d, J = 8.7 Hz), 7.50 (1 H, d, J = 8.7 Hz), 7.75 (2 H, d, J = 8.9 Hz), 7.97 (2 H, d, J = 8.9 Hz), 8.04 (1 H, s); ^13^C NMR (75.5 MHz, acetone-d_6_) *δ* 117.8, 120.6, 122.8, 124.4, 128.4, 130.2, 130.6, 133.0, 135.2, 138.3, 141.9, 160.3, 168.1; HRMS for C_14_H_10_O_2_NClF_3_S [M^+^ + H] calcd. 348.00674, found 348.00589.

**5-Chloro-2-hydroxy-*N*-(3-pentafluorosulfanylphenyl)benzamide (2c)**

Yield: 121 mg (0.33 mmol, 33%); colorless solid of m.p. 197 °C; *R*_f_ = 0.54 (ethyl acetate/*n*-hexane, 1:3); *υ*_max_(ATR)/cm^−1^ 3329, 3136, 1638, 1617, 1603, 1580, 1562, 1480, 1443, 1435, 1416, 1364, 1332, 1305, 1282, 1216, 1193, 1183, 1150, 1104, 1094, 933, 911, 883, 859, 826, 815, 805, 781, 766, 705, 680, 655; ^1^H NMR (300 MHz, acetone-d_6_) *δ* 7.03 (1 H, d, J = 8.9 Hz), 7.50 (1 H, d, J = 8.9 Hz), 7.6–7.7 (2 H, m), 8.0–8.1 (2 H, m), 8.40 (1 H, s), 10.15 (1 H, s), 11.82 (1 H, s); ^13^C NMR (75.5 MHz, acetone-d_6_) *δ* 117.5, 119.5, 120.7, 122.8, 124.3, 125.6, 128.3, 130.5, 135.3, 139.5, 154.4–154.9 (m), 160.6, 168.4; HRMS for C_13_H_10_O_2_NClF_5_S [M^+^ + H] calcd. 374.00354, found 374.00262.

#### 2.3.2. Synthesis of Ethanolamine Salts—Typical Procedure

The niclosamide derivative (0.29 mmol) was dissolved in EtOH (3 mL), and ethanolamine (19 µL, 0.31 mmol) was added. The reaction mixture was stirred for 1 h and then diluted with *n*-hexane. The formed precipitate was dried in a vacuum.

**5-Chloro-2-hydroxy-*N*-(4-pentafluorosulfanylphenyl)benzamide × ethanolamine (3a)**

Yield: 81 mg (0.19 mmol, 66%); colorless gum; ^1^H NMR (300 MHz, DMSO-d_6_) *δ* 2.7–2.8 (2 H, m), 3.5–3.6 (2 H, m), 6.42 (1 H, d, J = 8.9 Hz), 6.96 (1 H, d, J = 8.9 Hz), 7.62 (1 H, s), 7.7–7.9 (4 H, m); ^13^C NMR (75.5 MHz, DMSO-d_6_) *δ* 41.9, 58.9, 112.8, 118.4, 118.7, 120.6, 123.6, 126.7, 128.1, 132.1, 144.0, 145.8–146.2 (m), 166.4, 170.1; HRMS for C_13_H_10_O_2_NClF_5_S [M^+^ + H] calcd. 374.00354, found 374.00259; C_13_H_8_O_2_NClF_5_S [M^−^ − H] calcd. 371.98789, found 371.98883.

**5-Chloro-2-hydroxy-*N*-(4-trifluoromethylsulfanylphenyl)benzamide × ethanolamine (3b)**

Yield: 110 mg (0.27 mmol, 93%); colorless gum; ^1^H NMR (300 MHz, DMSO-d_6_) *δ* 2.6–2.7 (2 H, m), 3.4–3.5 (2 H, m), 6.37 (1 H, d, J = 8.9 Hz), 6.92 (1 H, d, J = 8.9 Hz), 7.5–7.6 (3 H, m), 7.78 (2 H, d, J = 8.7 Hz); ^13^C NMR (75.5 MHz, DMSO-d_6_) *δ* 40.1, 61.3, 112.1, 113.6, 118.4, 120.2, 123.7, 128.0, 131.8, 137.4, 143.9, 166.4, 170.6; HRMS for C_14_H_10_O_2_NClF_3_S [M^+^ + H] calcd. 348.00674, found 348.00594; C_14_H_8_O_2_NClF_3_S [M^−^ − H] calcd. 345.99109, found 345.99202.

### 2.4. Anticancer Activity

#### 2.4.1. Cell Lines and Culture Conditions

The FLO-1, SK-GT-4, and THP-1 cell lines were a gift from Dr. Shrikant Anant’s laboratory. Cells were grown in 10% fetal bovine serum (Fisher Scientific#FB12999102, Pittsburgh, PA, USA) and 1% antibiotic/antimycotic agent (Corning, Tewksbury, MA, USA) in RPMI 1640 media mixed with 25 µM HEPES, L-glutamine (Corning, MA, USA). The cell lines were grown in a 5% CO_2_ incubator at 37 °C and used below 15 passages for the experiments.

#### 2.4.2. Proliferation Assay

The proliferation assay used a 3-(4,5-dimethylthiazol-2-yl)-2,5-diphenyltetrazolium bromide (MTT) assay [41]. Briefly, 5000 FLO-1 and SK-GT-4 cells were counted and seeded in each well in 96-well plates. Post 24 h of incubation, EAC cells were treated with multiple concentrations (0–10 μM) of niclosamide (Cayman chemical#10649, Ann Arbor, MI, USA) and its analogs. At respective time points, 10 μL of stock MTT reagent (Millipore Sigma, Bedford, MA, USA) (3 mg/mL) was added to each well and incubated for 1–2 h under the dark at 37 °C. After incubation, the purple formazan crystals were dissolved in equal volumes of DMSO, and the absorbance of the resulting clear solutions was recorded at 570 nm using a microplate reader [42]. A CCK-8 (Abcam, Cat no. ab228554, Burlingame, CA, USA) assay was used for the THP-1 cells. Briefly, 5000 cells were plated in every well of a 96-well plate. After 24 h of plating, THP-1 cells were treated with niclosamide and compound **3a**. After the respective time point, an equal amount of CCK-8 reagent was pipetted to the wells and incubated for one hour in an incubator, and finally, the absorbance was recorded at 450 nm [43]. The percent change in the proliferation was calculated by comparing the cell viability of the treated groups to the untreated control cells.

#### 2.4.3. Colony Formation Assay

In total, 500 SK-GT-4 and FLO-1 cells were seeded in each well of 6-well plates before 24 h treatment of niclosamide analog **3a** IC_50_ concentration and an equivalent dose of niclosamide. The media was changed after 72 h with drug-free media to remove drug exposure and allow them to grow and form colonies for 10–12 days. At the end of the experiment, the colonies were washed using PBS three times, fixed with 10% formalin solution (Fisher Scientific#23-305510, Pittsburgh, PA, USA), and stained using a 1% crystal violet (Sigma-Aldrich#C6158, St. Louis, MO, USA) solution in 10% ethanol for 15 min. The colonies were then washed with water, dried at room temperature, counted, and imaged. The number of colonies in the treated cells was compared with untreated cells [44]. 

#### 2.4.4. Spheroid Formation Assay

In the ultra-low attachment plates (Corning, Lowell, MA, USA), 500 SK-GT-4 cells were seeded in each well with the spheroid media [DMEM medium supplemented with EGF (20 ng/mL), FGF (20 ng/mL), heparin (4 µg/mL), B27 (10 mL in 500 mL of 50×) (Invitrogen, Carlsbad, CA, USA), and antibiotic pen/strep (1%)]. After 24 h, spheroids were treated with **3a** (IC_50_ dose) and an equivalent dose of niclosamide. Spheroids were counted after 7 days, and images were taken [44].

#### 2.4.5. Western Blot Analysis [45]

A total of 500,000 FLO-1 and SK-GT-4 cells were seeded in 10 cm^2^ dishes. After 24 h, cells were incubated with a vehicle, niclosamide analog **3a** (IC_50_ concentration), and niclosamide (equivalent dose of **3a** IC_50_ concentration). Then, 72 h post-treatment with the compounds, the media were removed to eliminate the dead/floating cells and washed with PBS. The cells were then scraped using a cell scraper in the lysis buffer containing a protease/phosphatase inhibitor cocktail (Thermo Scientific# A32959, Rockford, IL, USA). Further cells were lysed and sonicated using a probe sonicator (Fisher Scientific Model no FB120). The resulting lysates were centrifuged at 6500 rpm and 4 °C for 10 min. The BSA (Thermo Scientific, Rockford, IL, USA) method was used to estimate total protein levels in the prepared cell lysates, which were diluted with lysis buffer mixed with 2× Laemmli buffer (Biorad#1610737, Hercules, CA, USA) and boiled for 5 min at 37 °C. Equal volumes of protein (50 μg) were used on the SDS-PAGE for separation using gel electrophoresis. Then, the gel was transferred to a PVDF membrane (Immobilon, Millipore, Bedford, MA, USA) using a Biorad’s transfer assembly for 2 h at 90 V. The membranes were removed, incubated in 5% milk powder dissolved in TBST for one hour, washed three times with TBST for 5 min each, and incubated with respective primary antibodies overnight at 4 °C on a shaker. The following day, the blots were washed with TBST three times for 5 min each and incubated with the respective anti-rabbit (Invitrogen#31460) and anti-mouse HRP (Invitrogen#31430) secondary antibodies for 1 h. The membranes were washed with TBST thrice for 7 min each and visualized by Amersham ECL Western Blotting Detection Reagent (Cytiva#RPN2106, Marlborough, MA, USA). The blots were developed using a chemiluminescence system ChemiDoc-XRS+ instrument (Biorad), and Biorad’s image lab program was used to prepare images. All primary antibodies were purchased from cell signaling technology (CST, Beverly, MA, USA), including BCL2 (CST cat no. 4223), BCL-XL (CST cat no. 2762), Bax (CST cat no. 2772), PARP (CST cat no. 9532), p-STAT3 (CST cat no. 4113s), and non-phospho-(Active)-β-Catenin (CST cat no. 8814S) antibodies, while GAPDH (G-9) (sc-365062) was obtained from Santa Cruz Biotech, Inc. (Santa Cruz, CA, USA). All antibodies were diluted in 5% BSA (Sigma-Aldrich#1003561707) in TBST at a 1:1000 dilution.

#### 2.4.6. Molecular Docking

AutoDock Vina 1.1.2 software from the Molecular Graphics Lab, Scripps Research Institute, http://vina.scripps.edu/ (accessed on 20 April 2024), was used for molecular docking of compounds **2a** and niclosamide with STAT3 (PDB: 6NJS) and β-catenin (PDB: 1JDH) [46,47,48]. Chain A was selected by removing ligands (SD36 from 6NJS) and other protein chains (hTCF4 from 1JDH) before the molecular docking. Default parameters were applied to STAT3 and β-catenin proteins, niclosamide, and its analog for docking using Autodock tools 1.5.7. Polar hydrogens and Kollman and Gasteiger charges were applied to proteins and ligands prior to the docking. A grid box was designed around the SH2 domain in the case of STAT3 and the TCF-4-binding site in the case of β-catenin. The best protein–ligand conformations (approximately 10) were determined with Lamarckian GA. The most stable ligand–protein complex was selected based on the interaction with key hydrogen bonds and the lowest binding energy. The drug–protein complex was visualized using Pymol, https://pymol.org/2/ (accessed on 20 April 2024) [49].

### 2.5. Antifungal Activity

#### 2.5.1. Compound Screening

The antifungal activity of the compounds was tested against a geographically and genetically diverse set of *M. mycetomatis* isolates. These included the MM55 strain from Sudan, strains I1, I3, and I11 from India, strain P1 from Mali, strain SO1 from Somalia, strain Peru72012 from Peru, and strain CBS247.48 whose country of origin was unknown. All isolates were originally obtained from patients and identified and maintained in the Erasmus Medical Centre, Rotterdam, The Netherlands. In vitro susceptibility testing was performed as reported previously [50,51]. The only difference was that tetrazolium compound 3-(4,5-dimethylthiazol-2-yl)-5-(3-carboxymethoxyphenyl)-2-(4-sulfophenyl)-2*H*-tetrazolium salt (MTS) was used as a viability dye in endpoint reading instead of 2,3-bis-(2-methoxy-4-nitro-5-sulfophenyl)-2*H*-tetrazolium-5-carboxanilide salt (XTT).

#### 2.5.2. Toxicity and Activity in *Galleria mellonella*

In vivo efficacy was determined in larvae of the greater wax moth *Galleria mellonella*. Since *G. mellonella* is an invertebrate, it is not subject to directive 2010/63/EU of the European law on animal testing. Larvae were housed in the dark in Petri dishes with Whatman paper. To determine the toxicity in *G. mellonella* larvae, test compounds were injected on three consecutive days at 24 h intervals in the last pro-leg of healthy larvae followed by monitoring of the survival for ten days. If no significant difference between the control and treated larvae was observed, the compound was considered nontoxic. Larvae were further used in infection studies to determine the in vivo activity of test compounds against *M. mycetomatis* according to our previously published protocol [52].

### 2.6. DMPK Analysis

Kinetic solubility was tested using 10 mM stock solutions of test compounds in DMSO followed by dilution with an aqueous buffer to a final concentration of 250 µM and vortexed at 900 rpm for 24 h. Then, the samples were filtered and analyzed by UHPLC (Shimadzu Nexera X2, Milton Keynes, UK). 

CHI (chromatographic hydrophobicity index) logD was determined according to previously published methods [53,54]. Test compounds were prepared as 0.5 mM solutions in 50:50 acetonitrile/water and analyzed by reversed-phase UHPLC (wavelength 254 nm). By plotting the retention time of a set of reference compounds against known CHI values, the CHI value of test compounds was calculated according to their retention time, and this was then further converted to CHI logD.

Liver microsomes were applied to study the microsomal clearance of test compounds. The test compound (0.5 µM) was incubated with female CD1 mouse liver microsomes (0.5 mg/mL, 50 mM potassium phosphate buffer, pH 7.4), and the reaction started with the addition of excess NADPH (8 mg/mL, 50 mM potassium phosphate buffer, and pH 7.4). Immediately, at time 0, then at 3, 6, 9, 15, and 30 min, an aliquot of the incubation mixture was removed and mixed with acetonitrile to stop the reaction. An internal standard (IS) was added to all samples, the samples were centrifuged to sediment precipitated protein, and the plates were then sealed prior to UPLC-MSMS analysis (Xevo TQ-S Micro, Waters^TM^, Milford, MA, USA). An exponential decay fit was created from the response ratio (compound peak area/IS peak area) and incubation time, and subsequently, Clint (mL/min/mg protein) was calculated by multiplying the rate constant (k) of the exponential decay curve and incubation conditions (incubation volume/mg protein added). 

HepG2 hepatoma cells were used for hepatotoxicity studies. Cells were provided by ECACC with a certificate of analysis confirming STR profiling and were mycoplasma tested every time new stabilates were made. Cells were screened utilizing a resazurin-based fluorescence assay (λ_ex_ = 540 nm, λ_em_ = 590 nm) in a 384-well plate format. HepG2 cells (1 × 10^5^ cells/mL) were incubated with the compound in a 10-point, 1 in 3 dilution series from a top concentration of 100 µM, for 72 h. After the 72 h incubation period, resazurin (0.27 mM in a cell culture medium) was added and the plates were incubated for a further 3–4 h before the fluorescent signal was measured using PHERAstar (BMG Labtech, Ortenberg, Germany). IC_50_ values were calculated from two independent experiments.

### 2.7. Statistical Analysis

GraphPad Prism 7 (GraphPad Inc., Boston, MA, USA) was applied for the log-rank test, one-way ANOVA tests, and IC_50_ calculations. A *p*-value smaller than 0.05 was considered to be significant. * *p* < 0.05 and ** *p* < 0.01.

## 3. Results

### 3.1. Chemistry

**Scheme 1:**
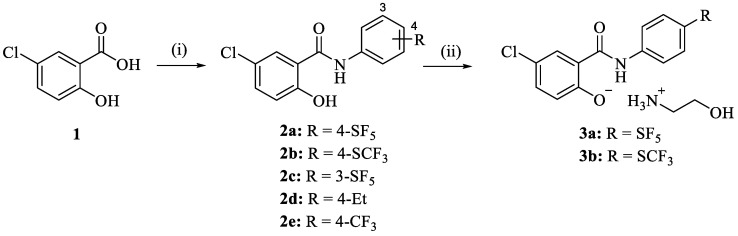
Reagents and conditions: (i) subst. aniline, EDCI, CH_2_Cl_2_, r.t., 24 h, 33–50%; (ii) ethanolamine, EtOH, r.t., 1 h, 66–93%.

The niclosamide derivatives **2a–e** were obtained from the reaction of 5-chlorosalicylic acid (**1**) with the respective substituted aniline. Compounds **2a** and **2b** were converted to their corresponding ethanolammonium salts **3a** and **3b** by reaction with ethanolamine in EtOH (Scheme 1). While **2d** and **2e** are known compounds prepared for comparison purposes in the following biological activity experiments, analogs **2a–c** and the phenolate salts **3a** and **3b** are new [39,40]. Compounds **2a–e** were obtained as solids, while the salts **3a** and **3b** were amorphous solids or gums. The ^1^H NMR spectra of **2a–e** displayed the aryl protons between 7.0 and 8.1 ppm. The ^13^C NMR spectra of **2a–c** showed the amide carbonyl and phenolic hydroxyl-carbon signals at 168.1–168.4 and 160.3–160.6 ppm, respectively. Some differences were observed in the NMR spectra of the ethanolamine salts **3a** and **3b**. Aryl protons of the salicyl ring showed highfield shifts in the ^1^H NMR spectra (e.g., 6.4 ppm for the most highfield aryl doublet signal), while the phenolate carbon (166.4 ppm) and the carbonyl signal (170.1–170.6 ppm) exhibited downfield shifts in the ^13^C NMR spectra when compared with the corresponding signals of their phenolic precursors **2a** and **2b**. In addition, proton (2.6–2.8, 3.4–3.6 ppm) and carbon signals (40.1–41.9 ppm, 58.9–61.3 ppm) of the ethanolamine component were observed in the NMR spectra of **3a** and **3b**. HR-MS detected the molecular ions of the new compounds **2a–c**, **3a**, and **3b**. Original spectra (NMR and ESI-MS) of the target compounds can be found in the Supplementary Materials (Figures S1–S19). 

### 3.2. Anticancer Activity

Compounds **2a–d**, **3a**, **3b**, and the parent drug niclosamide were initially tested for antiproliferative activity against SK-GT-4 and FLO-1 EAC cell lines (Table 1). 4-Pentafluorosulfanylanilide salt **3a** was the most active compound against both tested cell lines (IC_50_ = 1.03 µM for SK-GT-4 and 0.81 µM for FLO-1 cells) and slightly more active than niclosamide (IC_50_ = 1.77 µM for SK-GT-4 and 1.05 µM for FLO-1 cells) and cisplatin (IC_50_ = 2.28 µM for SK-GT-4 and 1.70 µM for FLO-1 cells) after 72 h. Compound **3a** exhibited promising sub-micromolar activity against FLO-1 cells. Compounds **2a** (4-pentafluorosulfanylanilide), **2b** (4-trifluoromethylanilide), and **2c** (3-pentafluorosulfanylanilide) as well as the salt **3b** conserved the anticancer activity of niclosamide, but they were slightly less active against FLO-1 cells than niclosamide. 4-Ethylanilide **2d** was the least active compound of this compound series. All compounds showed antiproliferative activities against EAC cell lines in the low micromolar concentration range. 

**Figure 2:**
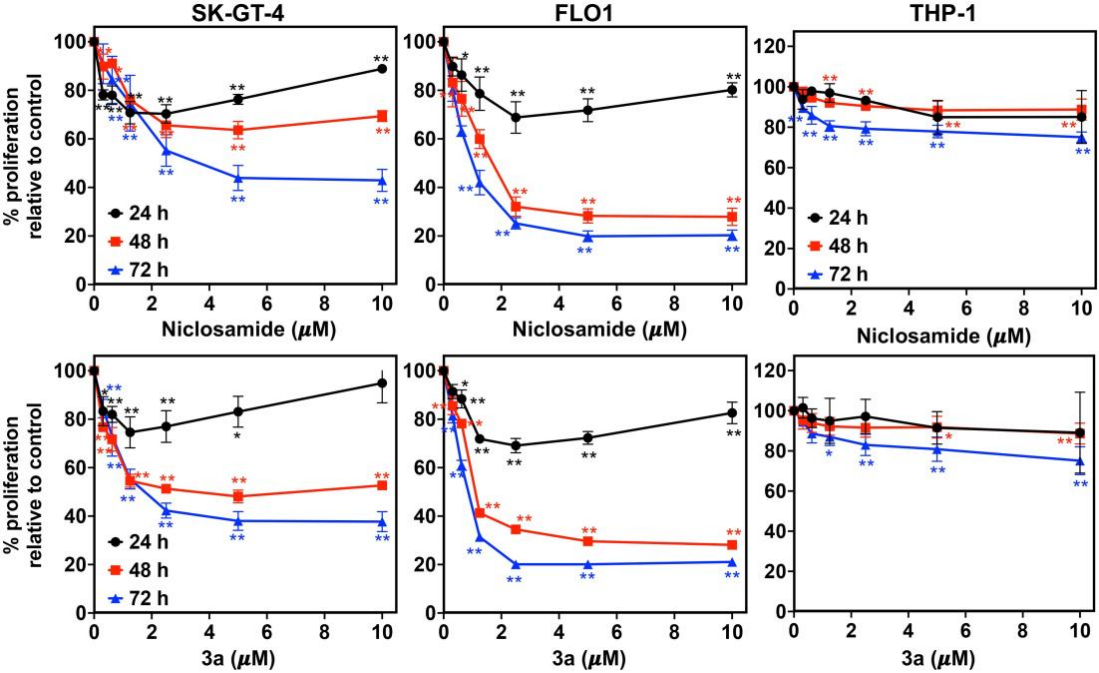
Time- and dose-dependent activity of niclosamide (upper row) and **3a** (bottom row) against EAC cell lines (SK-GT-4 and FLO-1) and THP-1 monocytes. * *p* < 0.05 and ** *p* < 0.01 as analyzed by one-way ANOVA test.

Compound **3a** exhibited dose- and time-dependent antiproliferative activities against SK-GT-4 and FLO-1 cells, analogously to niclosamide (Figure 2). While their activity was low after 24 h, it increased significantly after 48 h to reach a maximum activity after 72 h. In addition, **3a** did not affect the proliferation of non-malignant THP-1 monocyte cells (IC_50_ > 10 µM), which indicates a distinct tumor selectivity for this compound.

**Figure 3:**
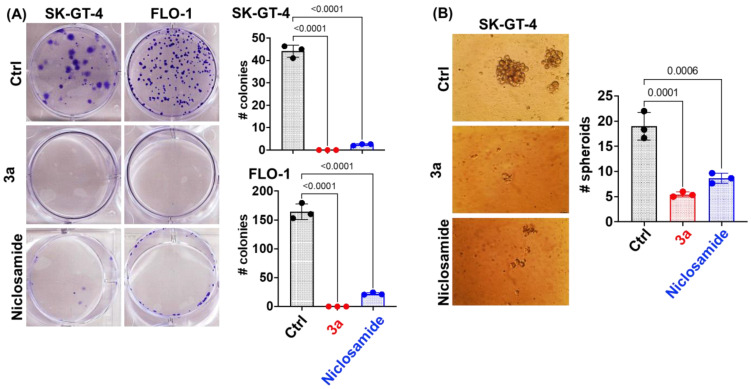
(**A**) Niclosamide and **3a** (1.5 and 0.8 µM for SK-GT-4 and FLO-1, respectively) inhibit colony formation by EAC cells at 48 h time point. (**B**) Niclosamide and **3a** (1.5 µM) inhibit spheroid formation by SK-GT-4 cells.

We used an IC_50_ concentration of **3a** at 48 h (~1.5 and ~0.8 µM for SK-GT-4 and FLO-1, respectively) and an equivalent concentration of niclosamide for further studies. Compound **3a** and niclosamide were tested for their long-term effect on the clonogenic potential of EAC cells (SK-GT-4 and FLO-1) using the colony formation assay (Figure 3A). Compound **3a** exhibited a complete inhibition of colony formation (size and number) in both EAC cell lines indicating an irreversible anticancer effect. Niclosamide was slightly less efficient than **3a**. The eradication of cancer stem cells (CSCs) is an attractive therapeutic strategy since they play a crucial role in EAC aggressiveness and resistance [55]. A spheroid formation assay was applied to evaluate the effects of **3a** and niclosamide on CSCs. Compound **3a** strongly inhibited spheroid formation by SK-GT-4 cells and outperformed niclosamide again (Figure 3B). 

**Figure 4:**
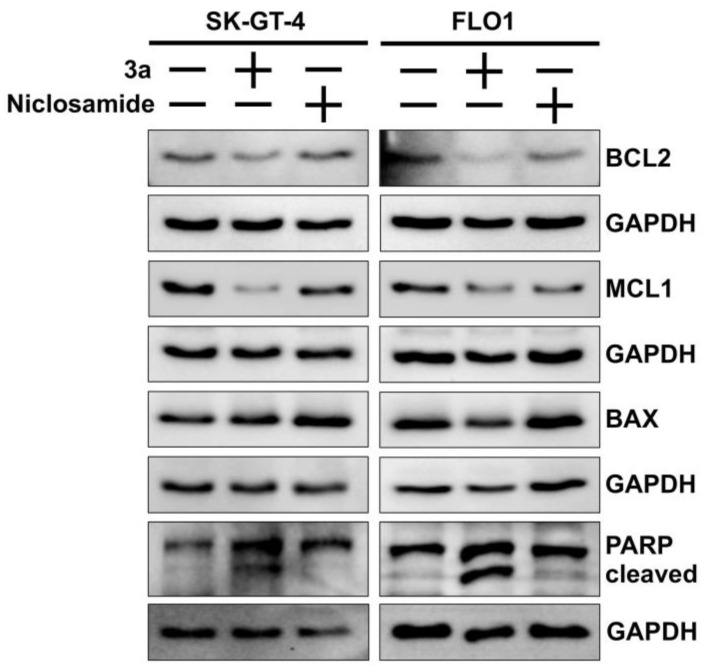
Effects of niclosamide and **3a** (1.5 and 0.8 µM for SK-GT-4 and FLO-1, respectively) on PARP cleavage, and expression of pro-apoptotic BAX and anti-apoptotic BCL2 and MCL1 in EAC cell lines. GAPDH (glyceraldehyde 3-phosphate dehydrogenase) served as control.

The colony formation and spheroid inhibition assays indicate a considerable induction of cancer cell death such as apoptosis by **3a**. Apoptosis is a form of programmed cell death, and anti-apoptotic mechanisms are a hallmark of resistant cancers [56]. Western blot analyses showed that the anti-apoptotic proteins BCL2 (B-cell lymphoma 2) and MCL1 (myeloid cell leukemia 1) were downregulated in EAC cells treated with **3a** while the expression of pro-apoptotic BAX (BCL2-associated X) did not differ from untreated cells (Figure 4). In addition, **3a** exhibited stronger PARP cleavage as a sign of apoptosis and a higher suppression of BCL2 and MCL1 than niclosamide in both EAC cell lines.

**Figure 5:**
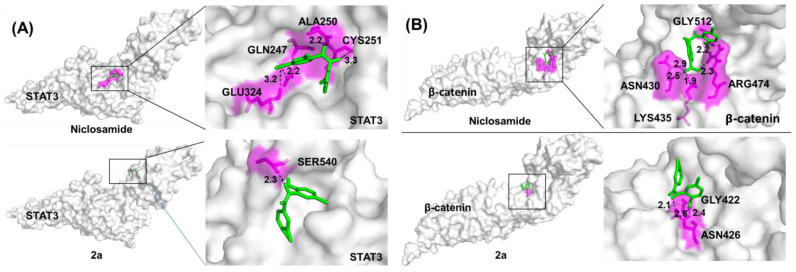
Molecular docking of niclosamide and **2a** (**A**) in the protein cavity of STAT3 and (**B**) in the protein cavity of β-catenin at the TCF4 binding site using the Autodock vina software program. Surface view of the binding mode. White: protein; pink: interacting amino acid; green: compound; black dotted line: H bond.

To identify possible protein targets, we carried out Autodock vina molecular docking calculations of **2a** (the phenolic precursor of the salt **3a**) and niclosamide bound to the protein structures of STAT3 and β-catenin, both of which are known niclosamide targets in cancer cells (Figure 5). To understand the interaction of compound **2a** and niclosamide in STAT3 and the β-catenin protein, we selected the SH2 domain and the TCF4-binding site, respectively. Compound **2a** bound to STAT3 and β-catenin with improved binding energies (7.2–7.3 kcal/mol) when compared with niclosamide (6.1–6.6 kcal/mol) indicating higher affinities of **2a** for both targets when compared with niclosamide (Table 2). Notably, the binding modes of niclosamide and **2a** differed considerably in both investigated protein targets. Both compounds were differently oriented in the binding cavities and formed H bonds with different amino acids of the binding sites of STAT3 and β-catenin (Figure 5, Table 2). In addition, **2a** required fewer H bonds than niclosamide to generate higher affinities for the binding sites.

**Figure 6:**
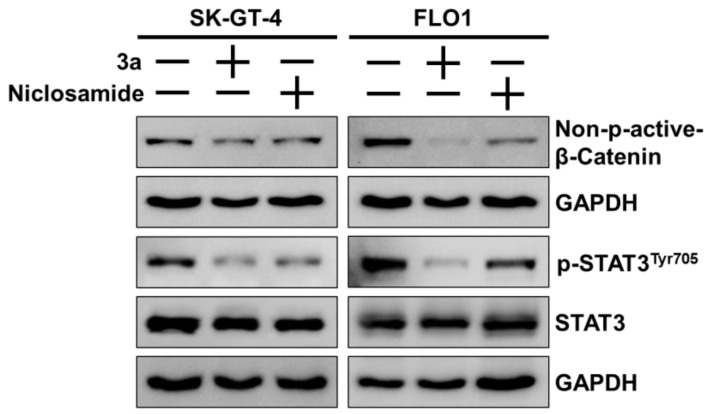
Effects of niclosamide and **3a** (1.5 and 0.8 µM for SK-GT-4 and FLO-1, respectively) on levels of p-STAT3^Tyr705^, STAT3, and (active) non-phospho-β-catenin in EAC cell lines. GAPDH (glyceraldehyde 3-phosphate dehydrogenase) served as control.

The STAT3 and β-catenin docking results were experimentally validated by Western blots in SK-GT-4 and FLO-1 cells treated with **3a** or niclosamide (Figure 6). Compound **3a** strongly suppressed activating the phosphorylation of STAT3 at TYR705 in both EAC cell lines without visible effects on the expression of STAT3. Niclosamide also inhibited activating STAT3 phosphorylation but not as strongly as **3a**. Likewise, niclosamide and, to a greater extent, **3a** suppressed active (non-phosphorylated) β-catenin in SK-GT-1 and FLO-1 cells, while these effects were more pronounced in FLO-1 cells than in SK-GT-4 cells.

### 3.3. Antifungal Activity

Initially, compounds **2a–e**, **3a**, and **3b** were tested for their growth inhibitory activity against the *M. mycetomatis* MM55 strain from Sudan (Table 3). Compounds **2a** and **2b** exhibited the highest activities against *M. mycetomatis* in the low nanomolar concentration range (IC_50_ = 0.3 µM and 0.2 µM, respectively). The corresponding ethanolamine salts **3a** and **3b** were slightly less active (IC_50_ = 0.9 µM each) than their phenol precursors. Compounds **2c** and **2e** showed similar activities (IC_50_ = 1.3 µM and 0.9 µM, respectively) while 4-ethylanilide **2d** was the least active compound of this series (IC_50_ = 6.6 µM). However, all tested niclosamide derivatives were distinctly more active than the parent drug niclosamide (IC_50_ = 15.0 µM, IC_90_ = 23.8 µM).

Compounds **2a–c** and **2e** were also tested for their activity (visualized as MIC values) against a panel of eight geographically and genetically diverse *M. mycetomatis* strains, and MIC50 values (medium inhibitory concentration, the concentration at which 50% of the isolates were completely inhibited) were determined thereof (Table 4). Again, **2a** and **2b** turned out to be the most active compounds with excellent MIC50 values of 0.5 µM. The Peru72012 strain turned out to be the most sensitive strain (IC_50_ = 0.3 µM for **2a** and **2b**). Of note, niclosamide (IC_50_ = 2.38 µM) and the 3-trifluoromethylanilide analog MMV665807 (IC_50_ = 9.93 µM) were reportedly less active than **2a** and **2b** (IC_50_ = 0.5 µM) against the Somalian SO1 isolate [28]. Compounds **2c** and **2e** were slightly less active (MIC = 1.0 µM) than **2a** and **2b**. However, **2c** was distinctly active against *M. mycetomatis* P1 (IC_50_ = 0.5 µM) and performed within the activity range of **2a** and **2b** in this strain. The lowest activities (i.e., IC_50_ = 2.0 µM for **2c** and **2e**) were found in I11, MM55, and CBS-247.48 strains.

**Figure 7:**
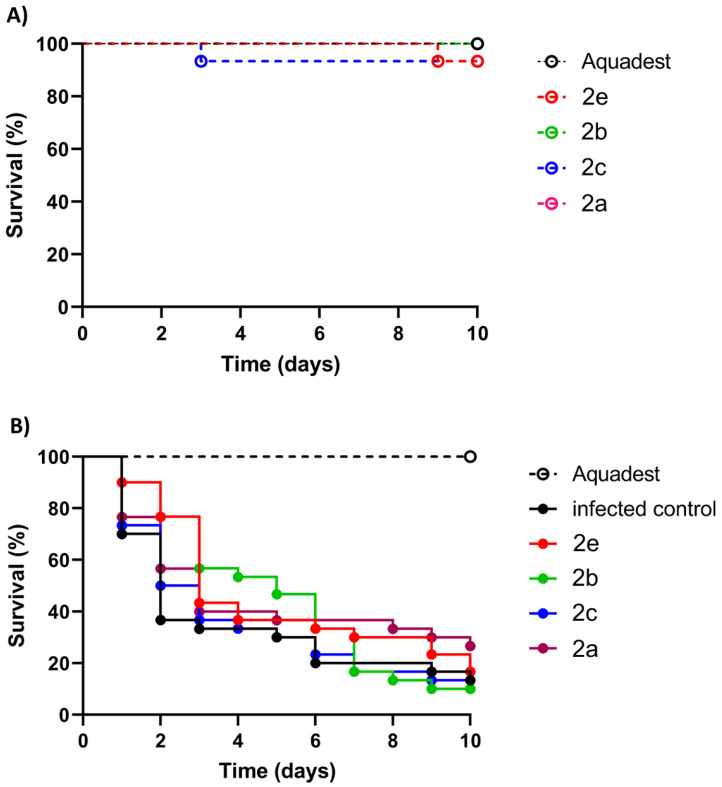
Evaluation of compounds **2a–c** and **2e** (20 µM) in *Galleria mellonella* larvae. (**A**) Toxicity evaluation of test compounds for 10 days indicated by larval survival (in %). (**B**) Activity of test compounds in *M. mycetomatis* MM55-infected *G. mellonella* larvae indicated by larval survival (in %).

The highly active compounds **2a–c** and **2e** were selected for in vivo activity testing using larvae of the wax moth *Galleria mellonella*, which is a well-established invertebrate animal model for the evaluation of fungal infections [57]. At first, the general toxicity of **2a–c** and **2e** to non-infected *G. mellonella* larvae at a high concentration of 20 µM was evaluated (Figure 7A). All tested compounds (**2a–c** and **2e**) were non-toxic and showed a promising toxicity profile because the survival curves were above 80% after compound injection. Thus, the compounds were suitable for in vivo experiments with *G. mellonella* larvae infected with the *M. mycetomatis* MM55 strain (Figure 7B, Table 5). Although a higher survival percentage was obtained in larvae treated with **2a** (26.7% vs. 13.33%), this difference was not statistically significant. Compounds **2b**, **2c**, and **2e** showed no increased survival after ten days. Although **2b** showed no improvement after ten days, a considerable temporary effect was observed between days three and six before its efficacy diminished. In terms of the AUC (area under the curve) values, **2a**, **2b,** and **2e** showed comparable effects; however, the increases were statistically not significant. Compound **2c** was the least effective compound in the in vivo assay with infected *G. mellonella* larvae. 

### 3.4. DMPK Analysis

To evaluate the drug-like properties of the most promising antifungal compounds **2a** and **2b**, DMPK (drug metabolism and pharmacokinetics) analyses were carried out (Table 6). The experimental logD values of **2a** and **2b** were between 3 and 5 indicating poor solubility and increased metabolic clearance, which was confirmed for **2b** by the microsomal clearance assays. Compound **2a**, however, showed a reasonable clearance and half-life (>30 min) in liver microsomes. HepG2 hepatoma cells of low tumorigenicity were used as a liver cell model for drug toxicity studies. Both **2a** and **2b** were toxic to HepG2 albeit less toxic than to EAC cells. Kinetic solubility experiments revealed low solubility for **2a** (0.67 µM) and stability issues for **2b**. 

## 4. Discussion

The new compounds **2a** and **2b** are potential drug candidates obtained by a simple synthetic procedure from commercially available starting materials. This enables large-scale syntheses and the mass production of these drug candidates which is of importance for future clinical studies and global applications [24,58]. Compounds **2a** and **2b** displayed improved activities against the fungus *M. mycetomatis* when compared with the activity of the parent compound niclosamide, an approved anthelminthic. In addition, the ethanolammonium salt **3a** revealed higher anticancer activities against EAC cell models when compared with the activities of niclosamide and cisplatin, and it was not toxic to non-malignant THP-1 cells. Thus, the replacement of the problematic nitro group of niclosamide by SF_5_ and SCF_3_ moieties conserved and even enhanced the anticancer and antifungal activities of niclosamide. Notably, a SF_5_ and SCF_3_ substitution of the anilide ring appeared superior to the ethyl and CF_3_ substitution of compounds **2d** and **2e**. The 3-pentafluorosulfanylanilide **2c** was less antifungal than **2a** and **2b** except for one strain (P1), but **2c** exhibited better anticancer activities against EAC than **2a** and **2b**. The described positive effects of SF_5_ and SCF_3_ groups on biological activities are in line with previously disclosed antimicrobial, antiparasitic, and anticancer compounds [34,35,36,37,38]. 

Given the limited arsenal of anticancer drugs available for the therapy of EAC, the superior activities of the new SF_5_-substituted salicylanilides in comparison with the performance of the standard drug cisplatin are promising [15,16]. A deeper investigation of the anticancer activity of salt **3a** against EAC cells revealed persistent long-term effects by blocking EAC cell colony formation and inhibiting EAC spheroid growth. The strong suppression of the anti-apoptotic proteins BCL2 and MCL1 indicates apoptosis induction as a mode of cell death in EAC cells treated with **3a**. Of note, BCL2 expression was correlated with EAC development, and the downregulation of MCL1 was associated with apoptosis induction in EAC cells [59,60]. Since cancer resistance to the BCL2 inhibitor venetoclax is based on the upregulation of alternative anti-apoptotic factors such as MCL1, the suppression of both BCL2 and MCL1 by **3a** appears especially promising [61]. The reason why salt **3a** is more anticancer-active than **2a** is unclear; however, in a study with liver cancer cells, niclosamide ethanolamine also showed slightly higher cytotoxicities than niclosamide [62].

Mechanistically, niclosamide is an inhibitor of Wnt/β-catenin and STAT3 signaling [17,18]. Molecular docking of salicylanilide **2a** revealed STAT3 and β-catenin as possible drug targets of its phenolate **3a**, which was confirmed in EAC cells by Western blotting experiments. Notably, **3a** turned out to be a stronger inhibitor of STAT3 and β-catenin than niclosamide. STAT3 signaling plays a vital role in EAC including FLO-1 cells and was connected with anti-apoptotic BCL2 and MCL1 expression levels [63,64]. In addition, β-catenin was found to be a crucial factor in FLO-1 cells and a valuable drug target in resistant EAC cells [65,66]. Oncogenic Wnt/β-catenin signaling is closely associated with drug resistance, cancer stemness, and tumor initiation [67]. Hence, small-molecule inhibitors of the Wnt/β-catenin pathway can potentially become valuable anticancer drugs [68].

Compound **3a** revealed a low toxicity to THP-1 monocytes. In vivo experiments with *G. mellonella* larvae exhibited generally low or even absent toxicities of compounds **2a–c** and **2e**. Despite the low toxicity, none of the compounds were able to prolong the survival of *M. mycetomatis*-infected larvae at the applied doses of 20 µM. Because of the low toxicity, higher doses than 20 µM and/or prolonged treatment might be applied in future studies to achieve better responses by infected larvae. A combination with other antifungals might also be a reasonable strategy to improve the therapeutic outcome. 

An important mode of action of niclosamide is based on its uncoupling properties accompanied by ATP depletion. Several azole drugs also decrease ATP levels via fungal cell membrane damage [69]. However, suppressed ATP levels as a sign of toxicity were documented in hepatocytes treated with itraconazole and fluconazole [70]. While no toxic effects on *G. mellonella* larvae and THP-1 cells were found, toxicity to HepG2 hepatoma cells was observed for **2a** and **2b** (IC_50_ = 2.3–3.0 µM), which was slightly lower than their toxic effects on EAC cells. However, the activity of **2a** and **2b** against HepG2 cells was distinctly lower than their activity against *M. mycetomatis* cells. It might indicate activity against liver cancer, though, since they were only slightly less active against HepG2 cells than a published SF_5_-substituted vorinostat analog (IC_50_ = 1.8 µM) which was designed as an anticancer agent [71]. Certain azoles such as ketoconazole and posaconazole were also toxic to HepG2 cells at higher concentrations, which was associated with reduced cellular ATP levels [72]. In liver microsomes, **2a** exhibited a reasonable stability and half-life which reminds us of the improved pharmacokinetics of a SF_5_-substituted mefloquine analog [35,36]. However, caution is advised for these data because of the low solubility of **2a**, which can cause issues with PK properties and analysis (e.g., a low MS response was found for **2a** in the clearance assay). The solubility problem can be solved by salt formulations such as **3a**. Compound **2b** was found to be rather unstable which might be attributed to the SCF_3_ substituent being sensitive to oxidation to sulfoxides [73]. How far such sulfoxides contribute to the biological activity of **2b** remains to be shown.

## 5. Conclusions

The synthesis of SF_5_- and SCF_3_-substituted niclosamide-type salicylanilides is straightforward, and the new compounds **2a–c** and **3a** revealed distinct anticancer and antifungal activities. Mechanistic studies in EAC cells showed considerable interference with BCL2- and MCL1-associated anti-apoptotic mechanisms and suppression of crucial oncogenic signaling pathways by inhibiting STAT3 and β-catenin. Unique binding modes of **3a** in STAT3 and β-catenin, which differed distinctly from niclosamide binding to these targets, were visualized by docking calculations. Together with low toxicities found in non-malignant monocytes and invertebrate animals, compounds such as **2a** and **3a** have great potential to become new anticancer and antifungal drugs in the future. Only a closer look at possible hepatotoxic effects seems to be necessary in advanced animal studies of these compounds with vertebrate models. 

## Figures and Tables

**Table 1:** Antiproliferative activity (IC_50_ values in µM) ^1^ of test compounds **2a–d**, **3a**, and **3b** against SK-GT-4 and FLO-1 EAC cells after 72 h. Niclosamide and cisplatin served as positive controls.

Compound	SK-GT-4 (µM)	FLO-1 (µM)
**Niclosamide**	1.77 ± 0.77	1.05 ± 0.28
**Cisplatin**	2.28 ± 0.14	1.70 ± 1.48
**2a**	1.80 ± 0.17	1.62 ± 0.55
**2b**	2.02 ± 0.14	2.20 ± 0.36
**2c**	1.38 ± 0.45	1.38 ± 0.40
**2d**	7.10 ± 0.75	6.13 ± 0.96
**3a**	1.03 ± 0.10	0.81 ± 0.21
**3b**	1.98 ± 0.08	2.63 ± 0.46

^1^ Values represent means ± SD of at least three independent experiments.

**Table 2:** Docking scores for the binding of niclosamide and **2a** to STAT3 and β-catenin determined with Autodock vina.

Protein/Compd.	B.E. (kcal/mol)	H Bonds	Amino Acid	Distance (Å)
**STAT3**				
**Niclosamide**	−6.6	4	ALA250 CYS251 GLN247 GLU324	2.2 3.3 2.2 3.2
**2a**	−7.3	1	SER540	2.3
**β-Catenin**				
**Niclosamide**	−6.1	5	ASN430 LYS435 ARG474 GLY512	2.5 and 2.9 1.9 2.3 2.2
**2a**	−7.2	3	GLY422 ASN426	2.6 2.1 and 2.4

**Table 3:** Antifungal activities (IC_50_ and IC_90_ values in µM) ^1^ of niclosamide and test compounds **2a–e**, **3a**, and **3b** against *M. mycetomatis* MM55 strain.

Compound	IC_50_	IC_90_
**Niclosamide**	15.0 ± 1.6	23.8 ± 0.1
**2a**	0.3 ± 0.1	1.9 ± 0.6
**2b**	0.2 ± 0.1	0.5 ± 0.6
**2c**	1.3 ± 0.7	2.0 ± 0.1
**2d**	6.6 ± 2.0	8.4 ± 1.1
**2e**	0.9 ± 0.2	1.1 ± 0.2
**3a**	0.9 ± 0.1	1.3 ± 0.3
**3b**	0.9 ± 0.1	1.1 ± 0.2

^1^ Values represent means ± SD of at least three independent experiments.

**Table 4:** Antifungal activities (MIC values in µM) of **2a–c** and **2e** against eight *M. mycetomatis* strains and corresponding MIC50 values.

Strain/Compound	2a	2b	2c	2e
**MM55**	0.5	0.5	2.0	1.0
**SO1**	0.5	0.5	1.0	1.0
**I1**	0.5	0.5	1.0	1.0
**I3**	0.5	0.5	1.0	1.0
**I11**	0.5	0.5	2.0	2.0
**P1**	0.5	0.3	0.5	1.0
**Peru72012**	0.3	0.3	1.0	1.0
**CBS247.48**	0.5	0.5	2.0	2.0
**MIC50**	0.5	0.5	1.0	1.0

**Table 5:** *G. mellonella* larval survival data after 10 days (d10) for *M. mycetomatis*-infected larvae treated with **2a–c** and **2e** (20 µM). AUC, the area under the curve (associated graphs, see Figure 2). Control, untreated infected *G. mellonella* larvae.

Compd.	d10 Survival (%)	d10 Survival Corrected with Control (%)	d10 AUC	d10 ΔAUC	d10 *p*-Value (Log-Rank)	Significance
**2a**	26.67	13.33	445	113.3	0.1896	no
**2b**	10	−3.33	451.7	120	0.328	no
**2c**	10	−3.33	346.7	15	0.9951	no
**2e**	16.67	3.33	453.3	121.6	0.161	no
**Control**	13.33	-	331.7	-	-	-

**Table 6:** Drug metabolism and pharmacokinetics (DMPK) study of **2a** and **2b** including logD values, microsomal clearance and half-life, and cytotoxic activity against HepG2 hepatoma cells (IC_50_ values from two independent experiments) using resazurin assay.

Compd.	CHI logD	Microsomal Clearance (mL/min/mg Protein)	Microsomal T ½ (min)	HepG2 (µM)
**2a**	3.9	0.02	>30	2.26 ± 0.31
**2b**	4.3	0.21	6.66	2.99 ± 0.11

## Data Availability

The original data are available from the authors upon request. Antifungal activities of test compounds against *M. mycetomatis* can also be found on the Mycetoma Open Source/MycetOS website, https://github.com/OpenSourceMycetoma (accessed on 30 April 2024).

## Supplementary Materials

The following supporting information can be downloaded at: https://www.mdpi.com/article/10.3390/biomedicines12071621/s1, Figures S1–S12: ^1^H and ^13^C NMR spectra; Figures S13–S19: ESI-HRMS spectra.
