# Supplementary material for: Superior Anticancer and Antifungal Activities of New Sulfanyl-Substituted Niclosamide Derivatives
Source: Biomedicines. 2024 Jul 21;12(7):1621. doi: 10.3390/biomedicines12071621 (PMC11275179; doi:10.3390/biomedicines12071621)
Supplement: Supplementary file 1 [file biomedicines-12-01621-s001.zip › biomedicines-3073086-supplementary.pdf]

-Supporting Information-

Superior Anticancer and Antifungal Activities of New Sulfanyl-Substituted Niclosamide Derivatives

Jingyi Ma [1,†], Dileepkumar Veeragoni [2,†], Hindole Ghosh [2], Nicole Mutter [3], Gisele Barbosa [3], Lauren Webster [3], Rainer Schobert [4], Wendy van de Sande [1,*], Prasad Dandawate [2,*] and Bernhard Biersack [4,*]

[1] Department of Medical Microbiology and Infectious Diseases, Erasmus MC, University Medical Center Rotterdam, Dr. Molewaterplein 40, 3015 GD Rotterdam, The Netherlands; m.jingyi@erasmusmc.nl

[2] Department of Cancer Biology, University of Kansas Medical Center, 3901 Rainbow Boulevard, Kansas City, KS 66160, USA; dveeragoni@kumc.edu (D.V.); hghosh@kumc.edu (H.G.)

[3] Wellcome Centre—Antiinfectives Research, School of Life Sciences, University of Dundee, Nethergate, Dundee DD1 4HN, UK; n.l.mutter@dundee.ac.uk (N.M.); bgiselejf@gmail.com (G.B.); l.a.webster@dundee.ac.uk (L.W.)

[4] Organic Chemistry Laboratory, University Bayreuth, Universitätsstrasse 30, 95440 Bayreuth, Germany; rainer.schobert@uni-bayreuth.de

* Correspondence: w.vandesande@erasmusmc.nl (W.v.d.S.); pdandawate@kumc.edu (P.D.); bernhard.biersack@yahoo.com (B.B.)

† These authors contributed equally to this work.

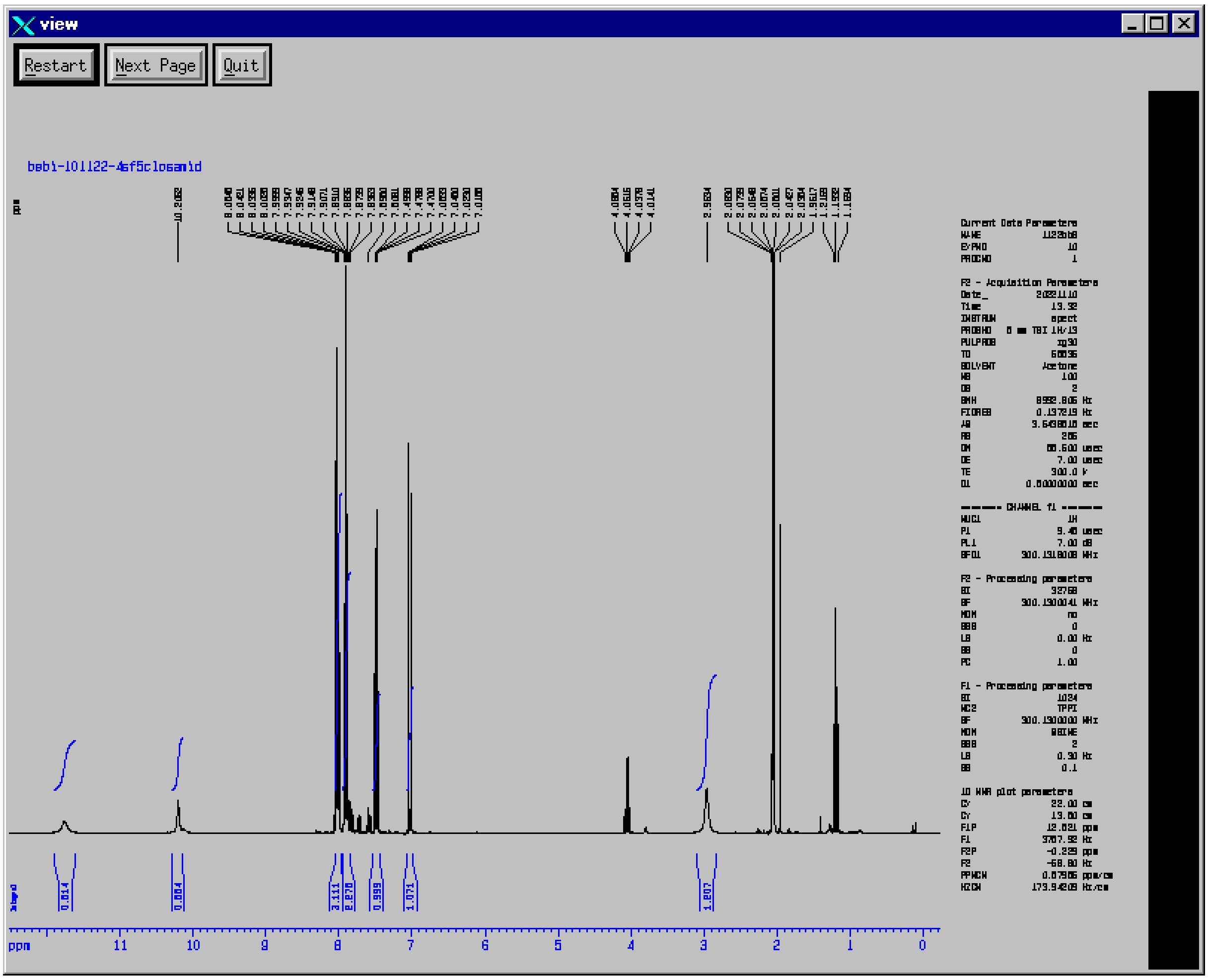

Figure S1. ^{1}H NMR spectrum of **2a**.

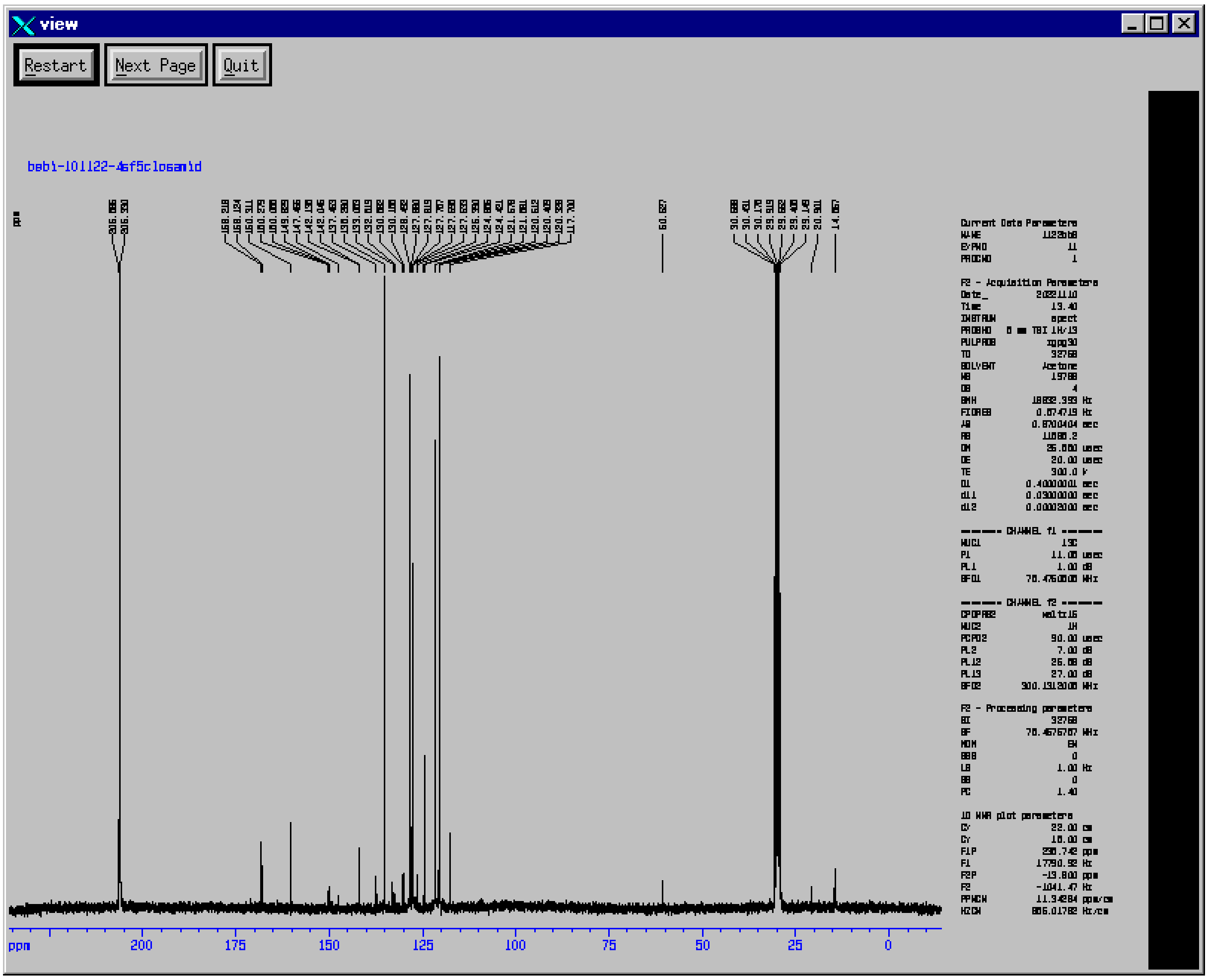

Figure S2. ^{13}C NMR spectrum of **2a**.

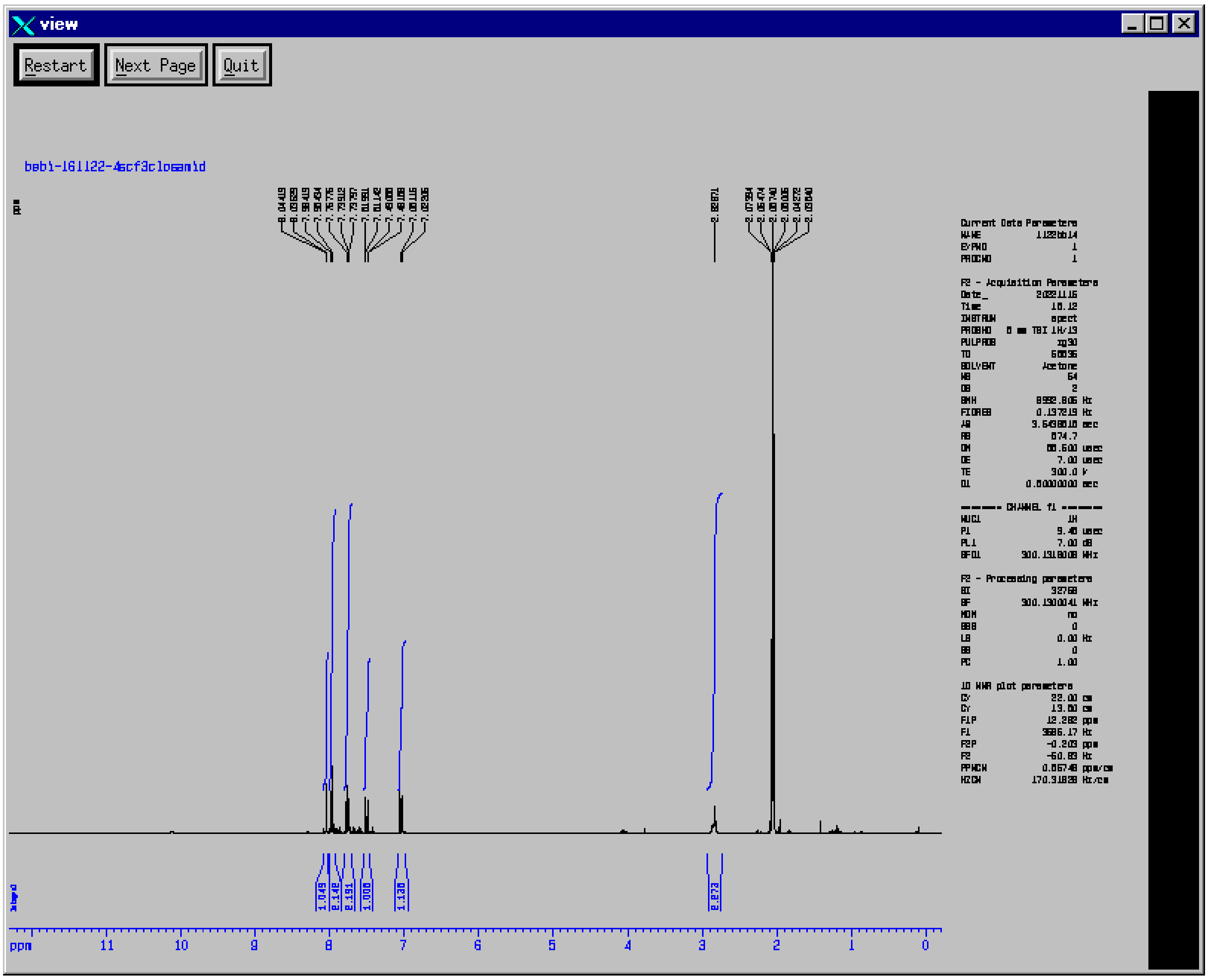

Figure S3. ^{1}H NMR spectrum of **2b**.

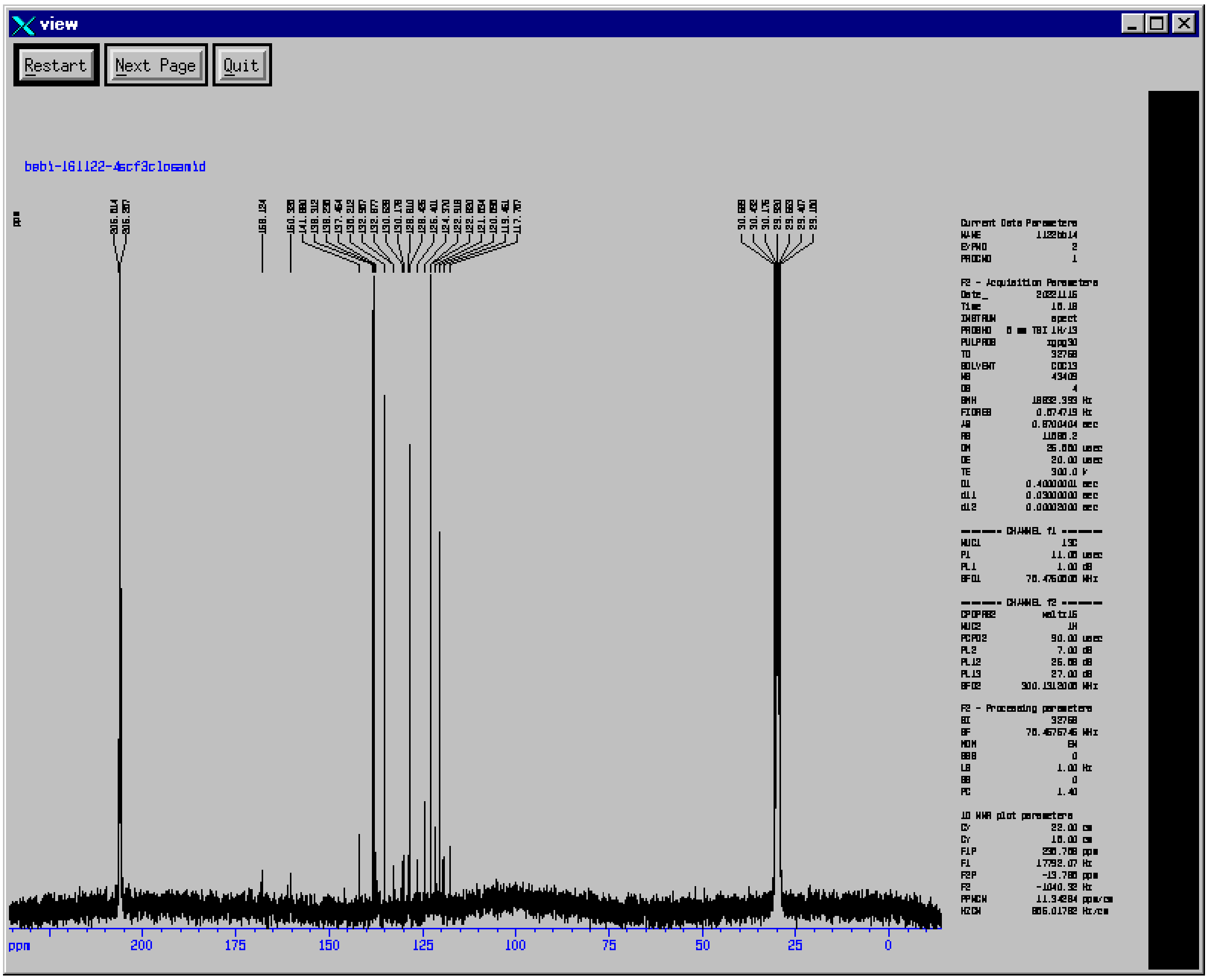

Figure S4. ^{13}C NMR spectrum of **2b**.

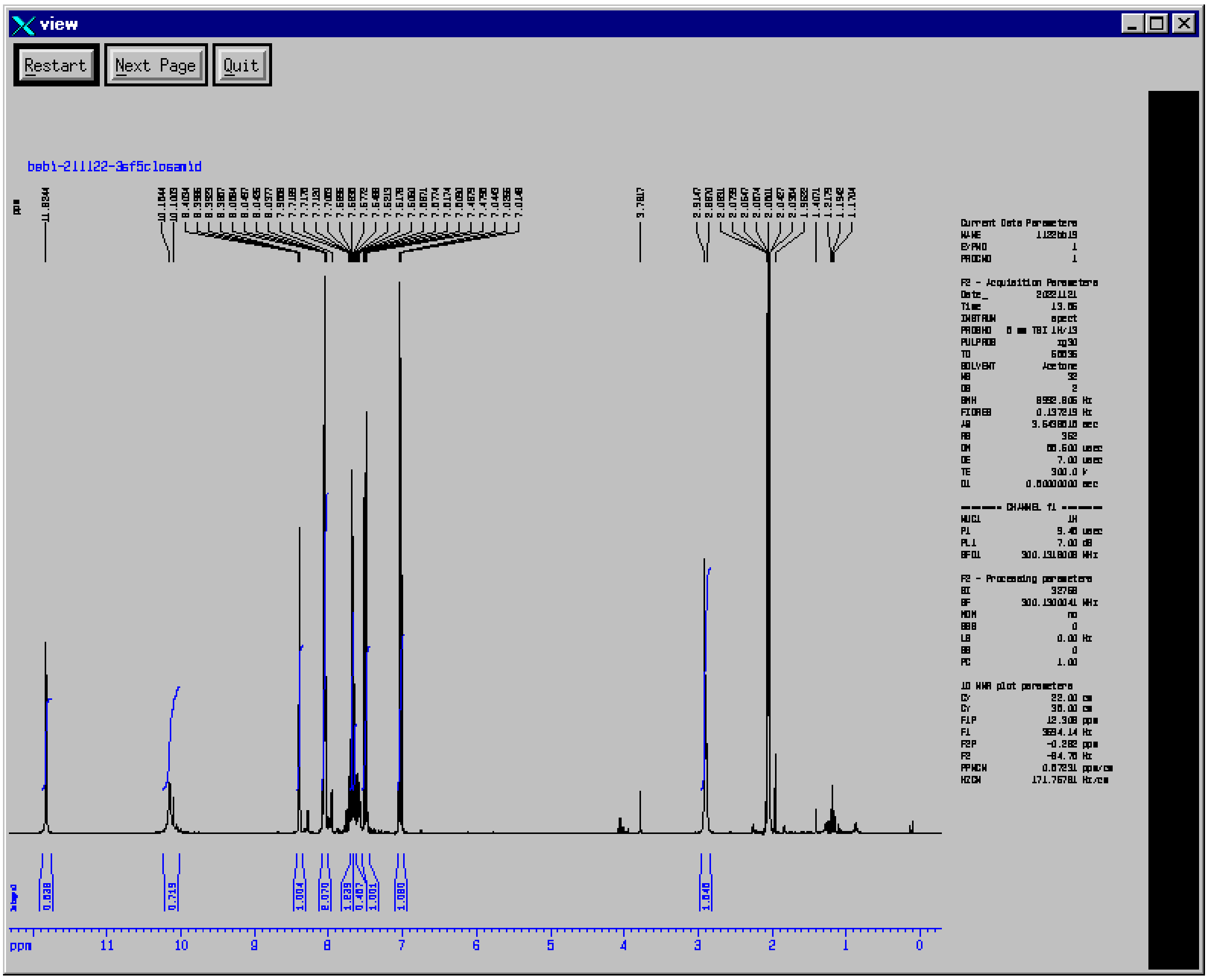

Figure S5. ^{1}H NMR spectrum of **2c**.

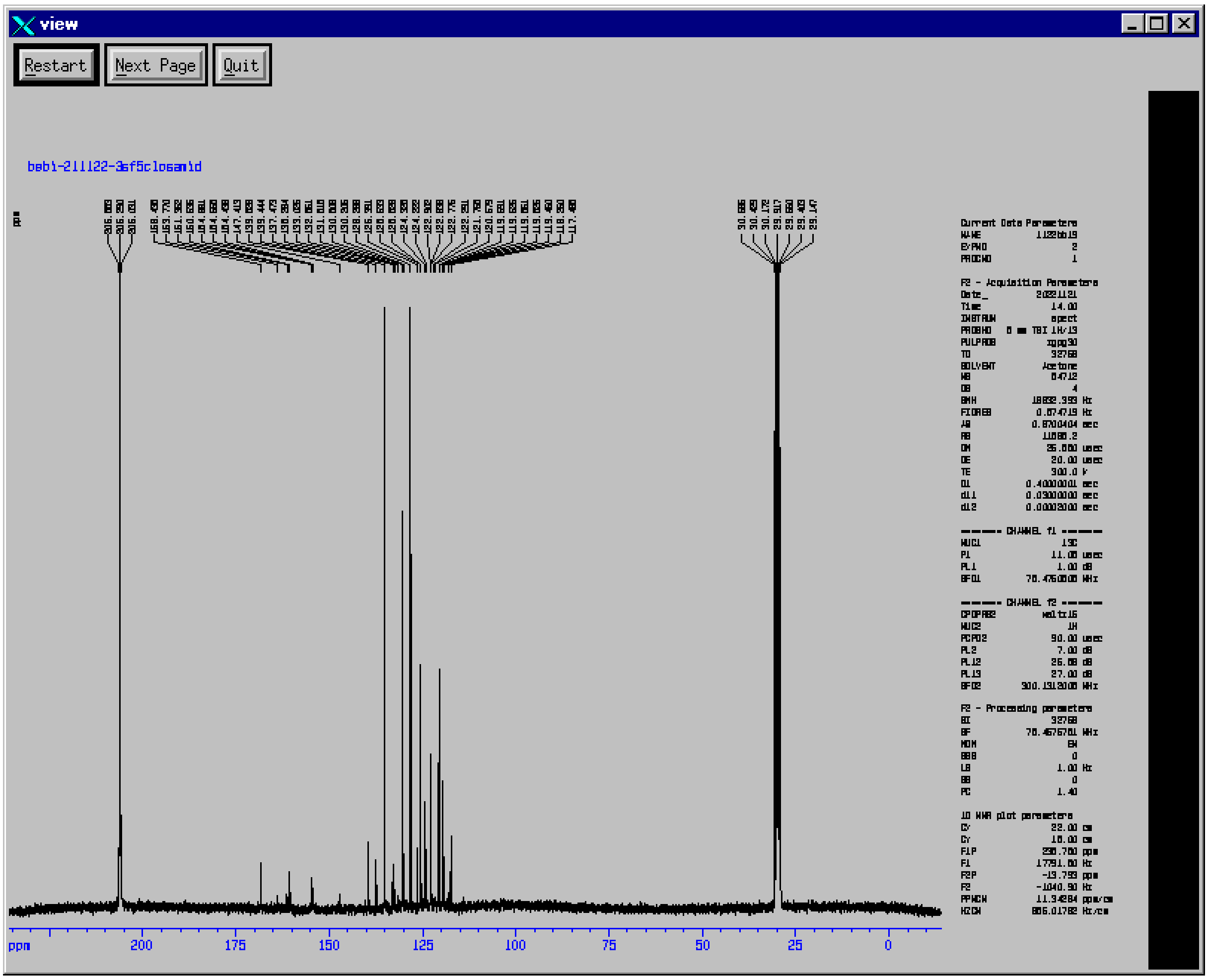

Figure S6. ^{13}C NMR spectrum of **2c**.

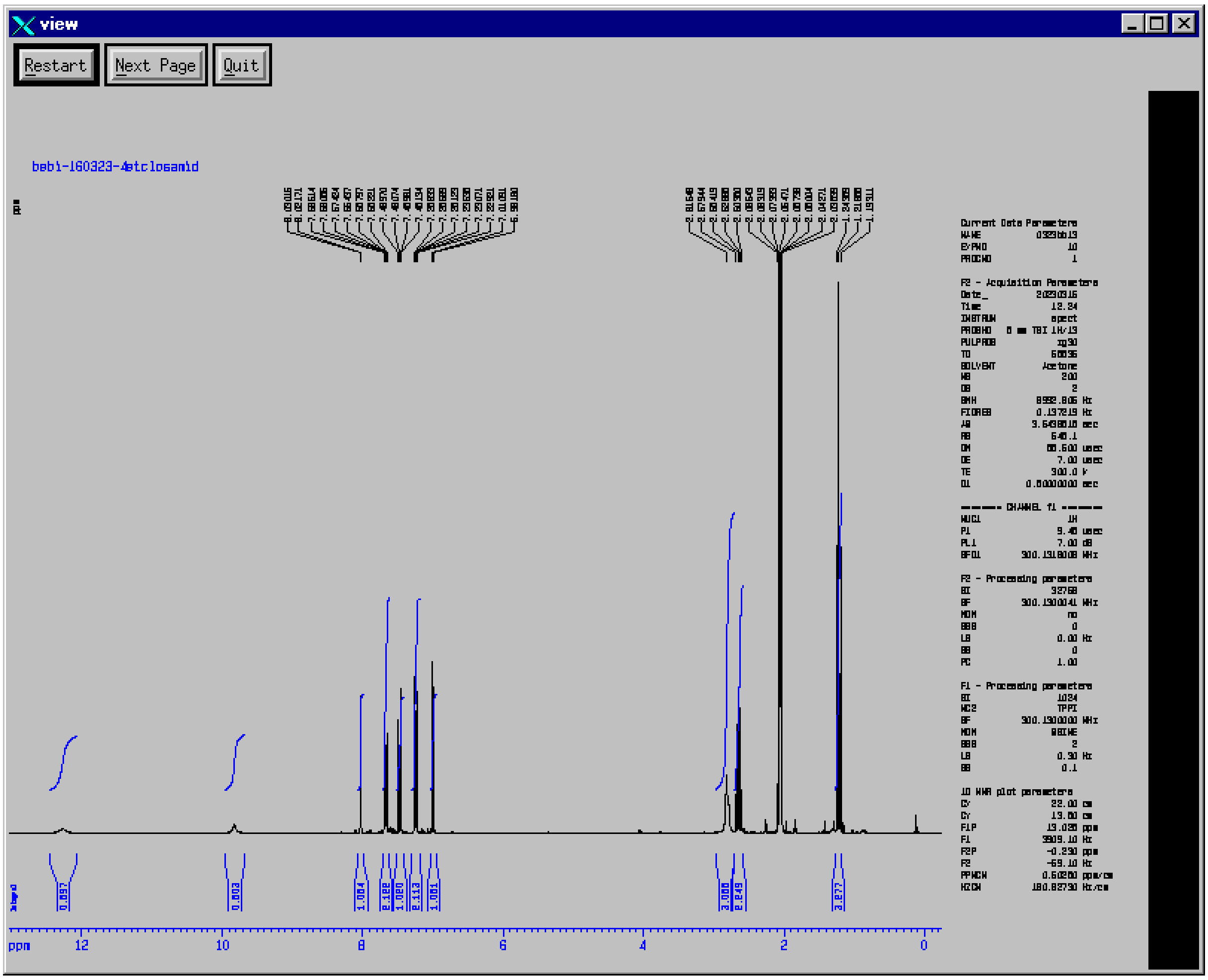

Figure S7. ^{1}H NMR spectrum of **2d**.

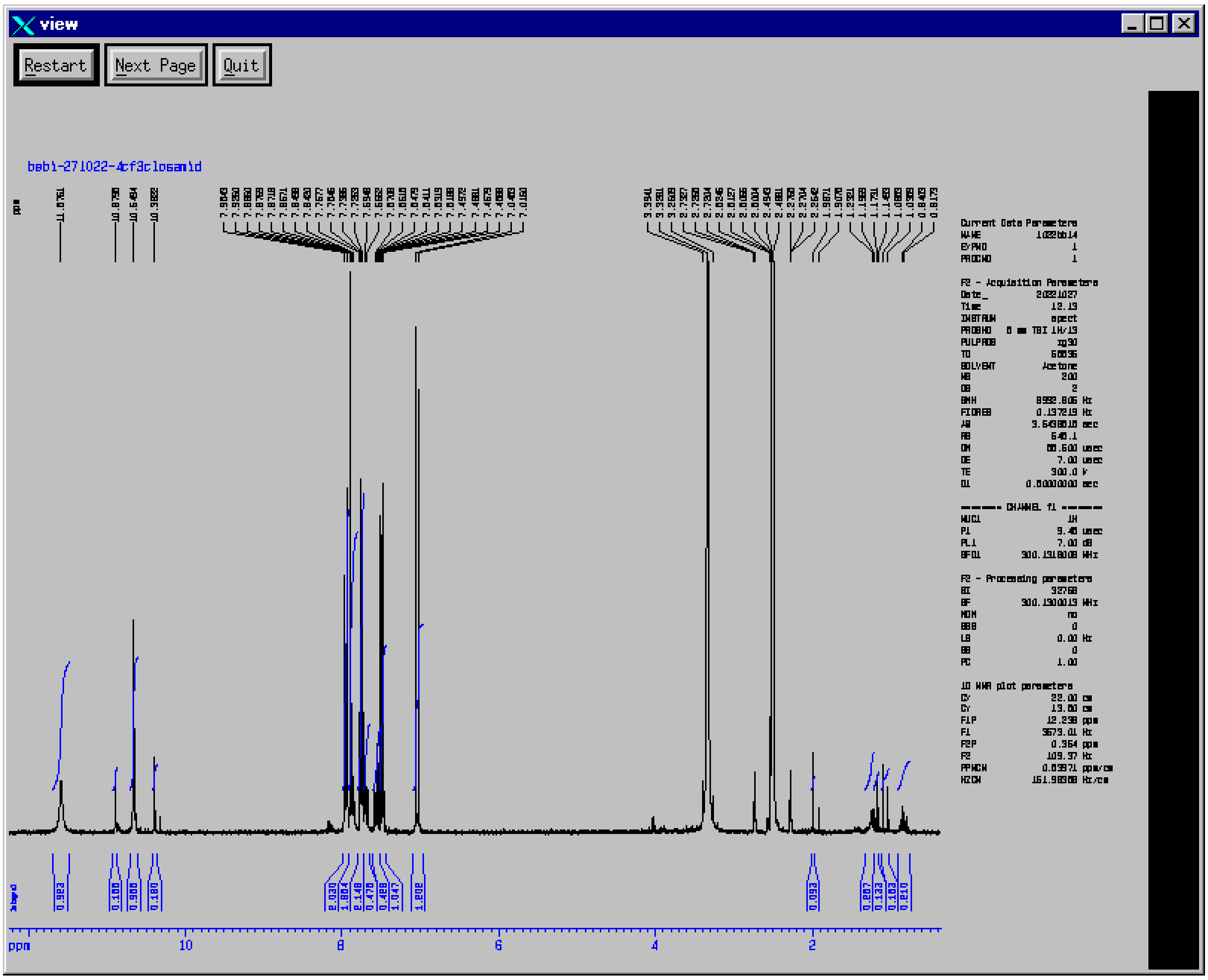

Figure S8. ^{1}H NMR spectrum of **2e**.

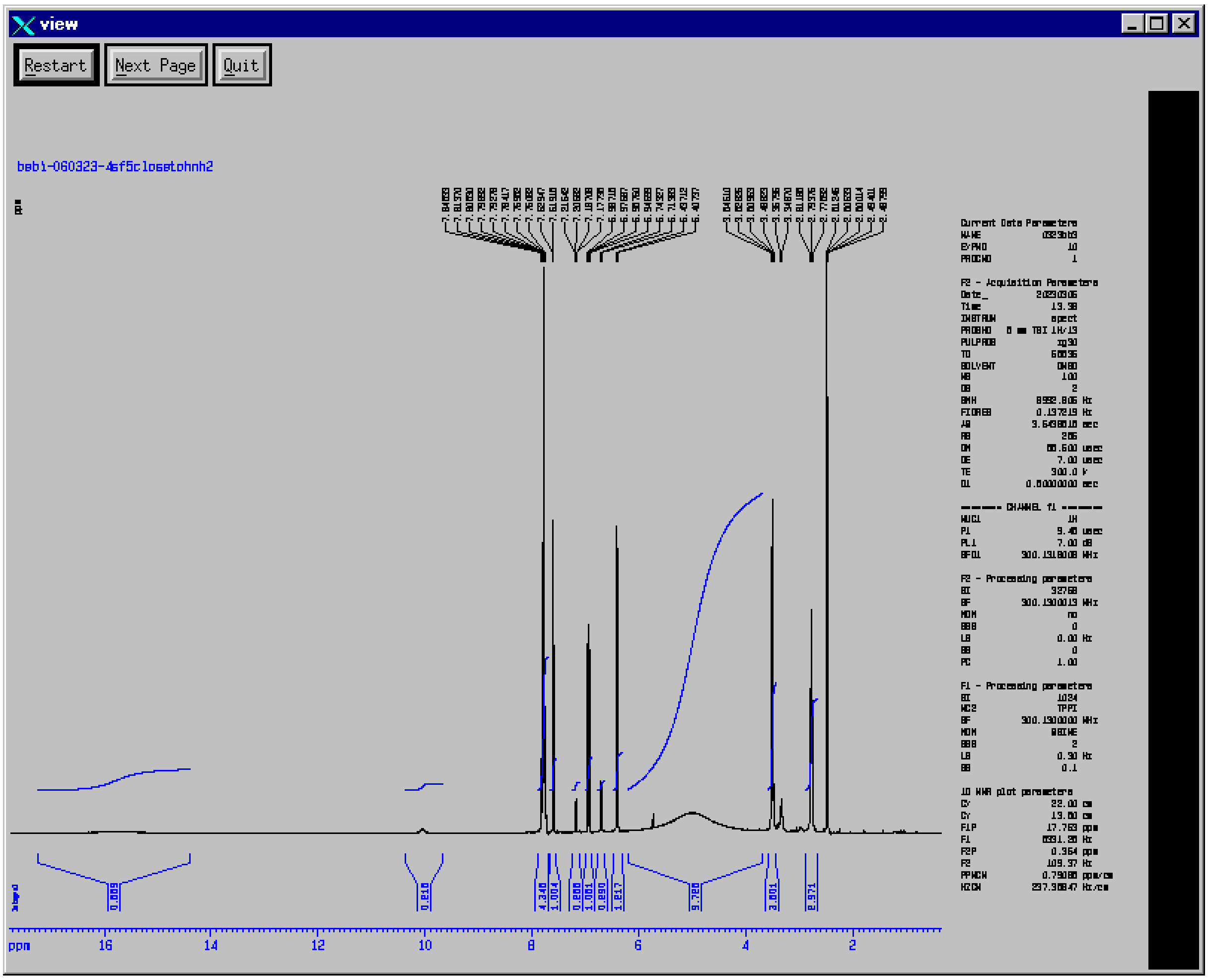

Figure S9. ^{1}H NMR spectrum of **3a**.

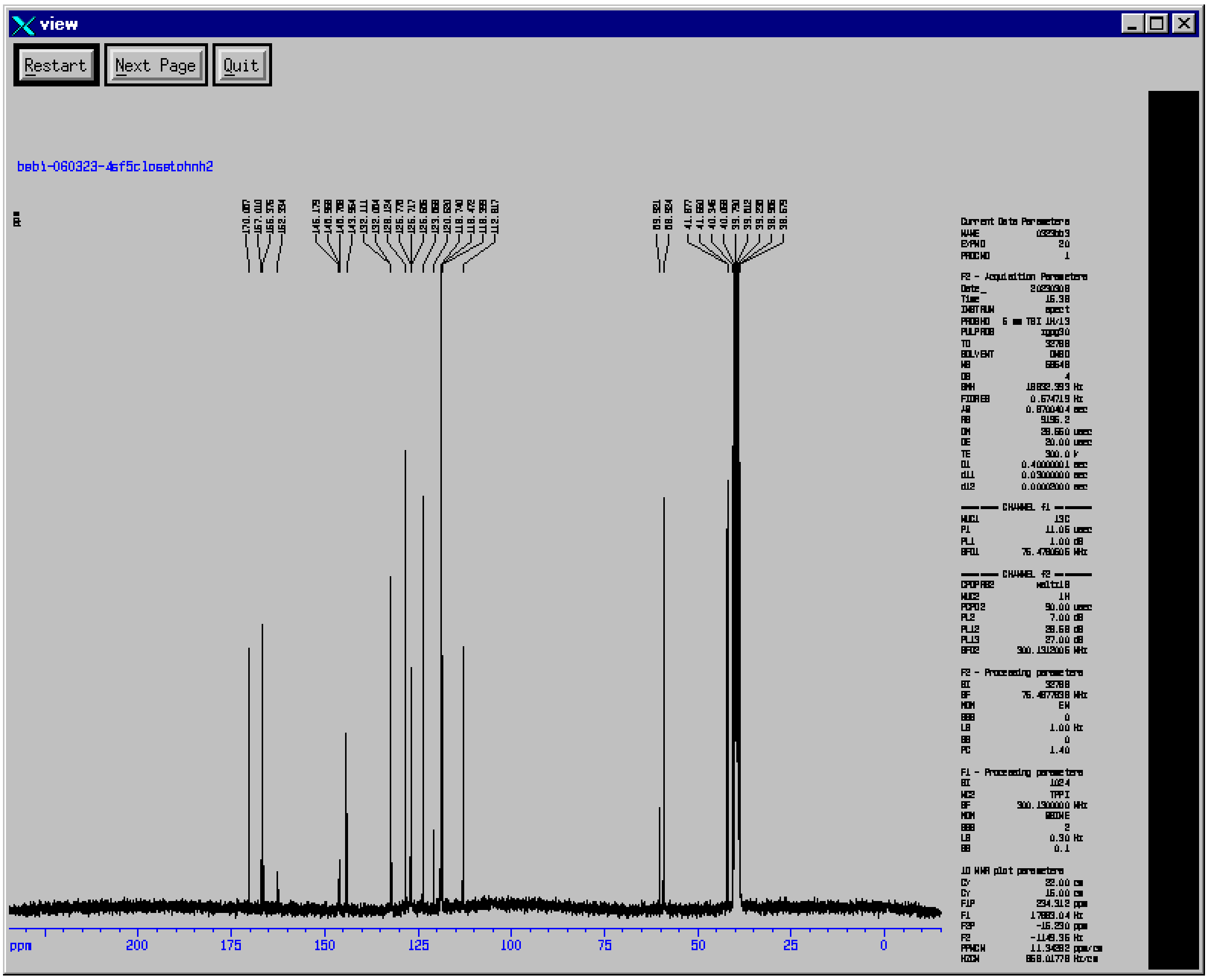

Figure S10. ^{13}C NMR spectrum of **3a**.

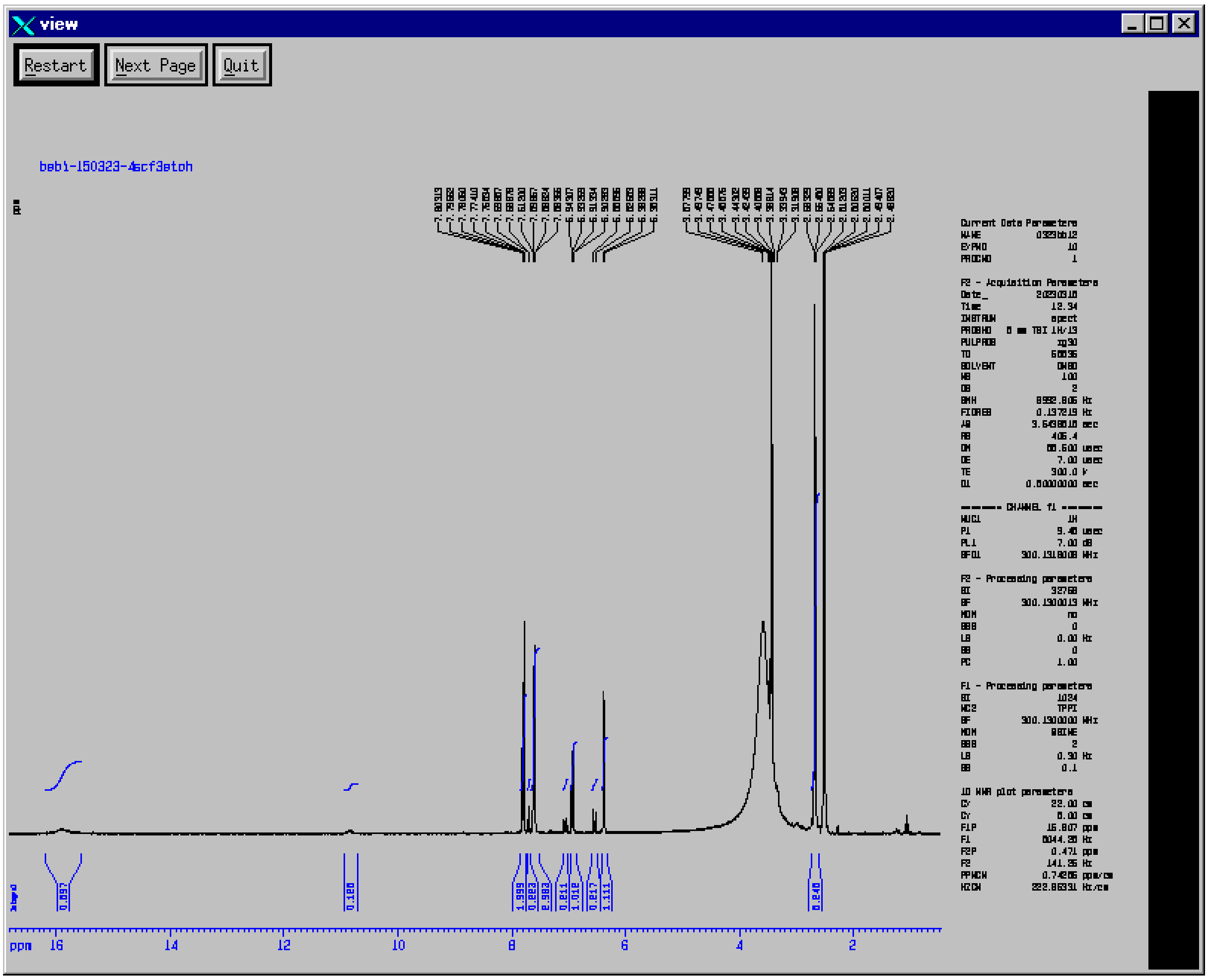

Figure S11. ^{1}H NMR spectrum of **3b**.

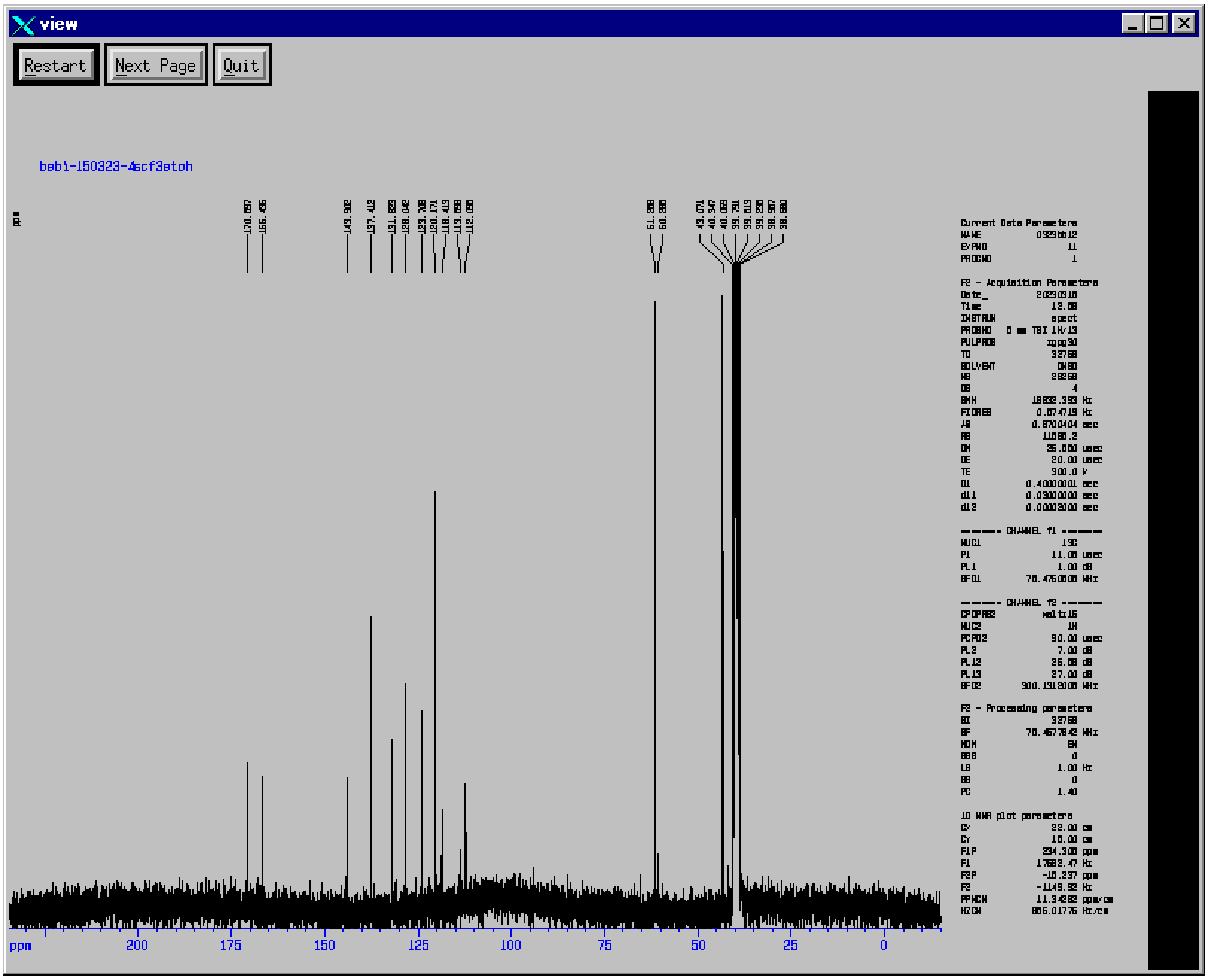

Figure S12. ^{13}C NMR spectrum of **3b**.

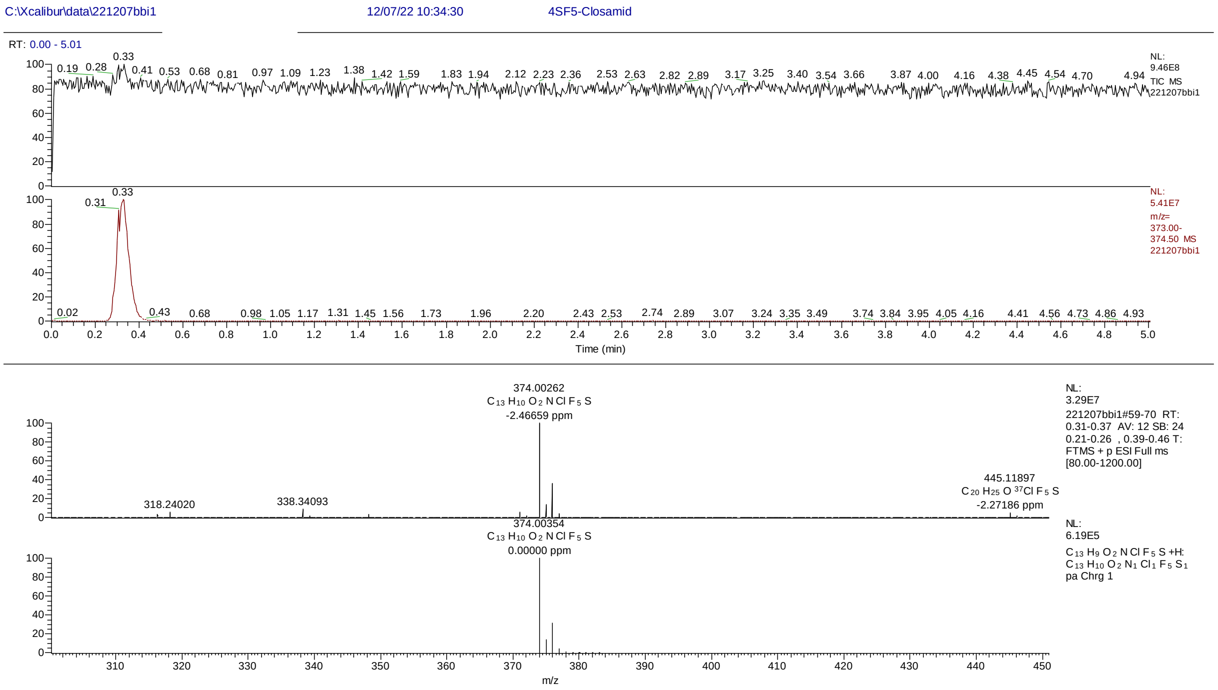

Figure S13. ESI-HRMS spectrum of **2a**.

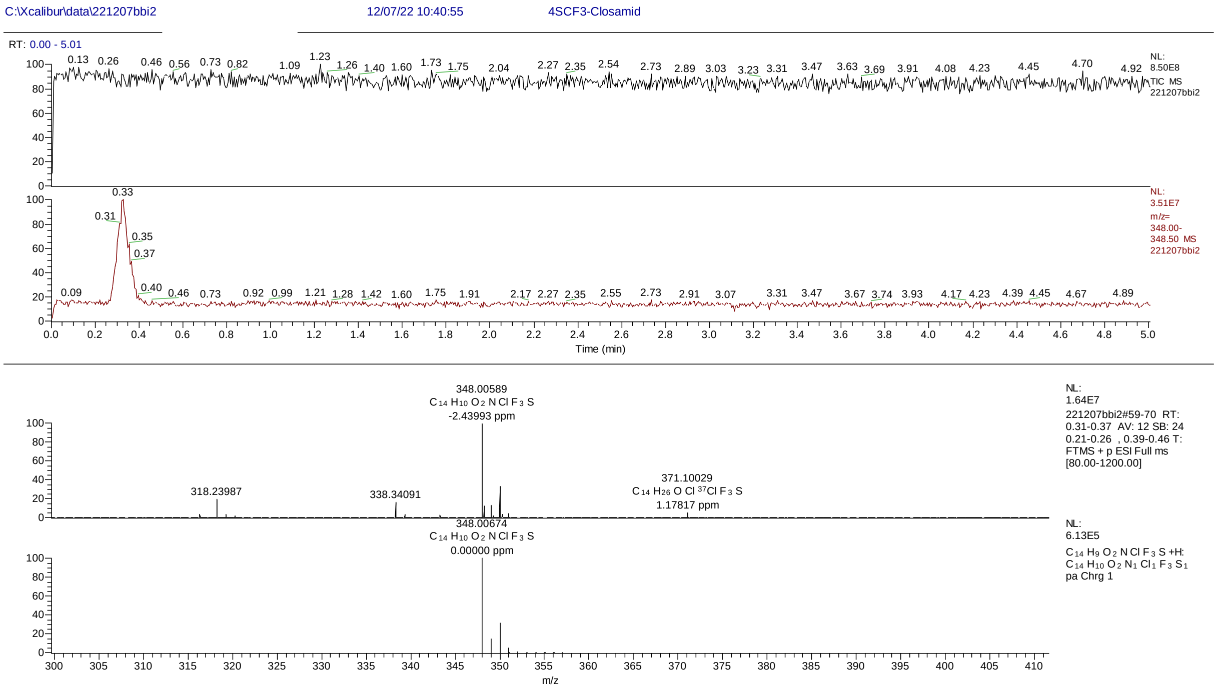

Figure S14. ESI-HRMS spectrum of **2b**.

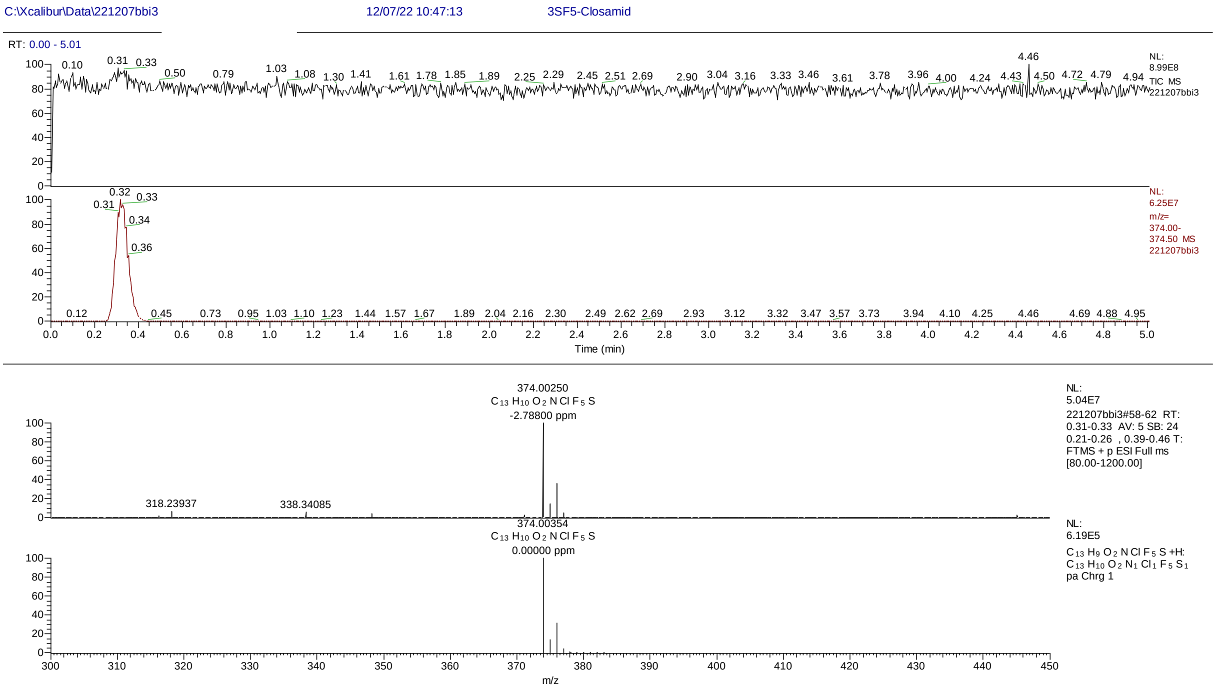

Figure S15. ESI-HRMS spectrum of **2c**.

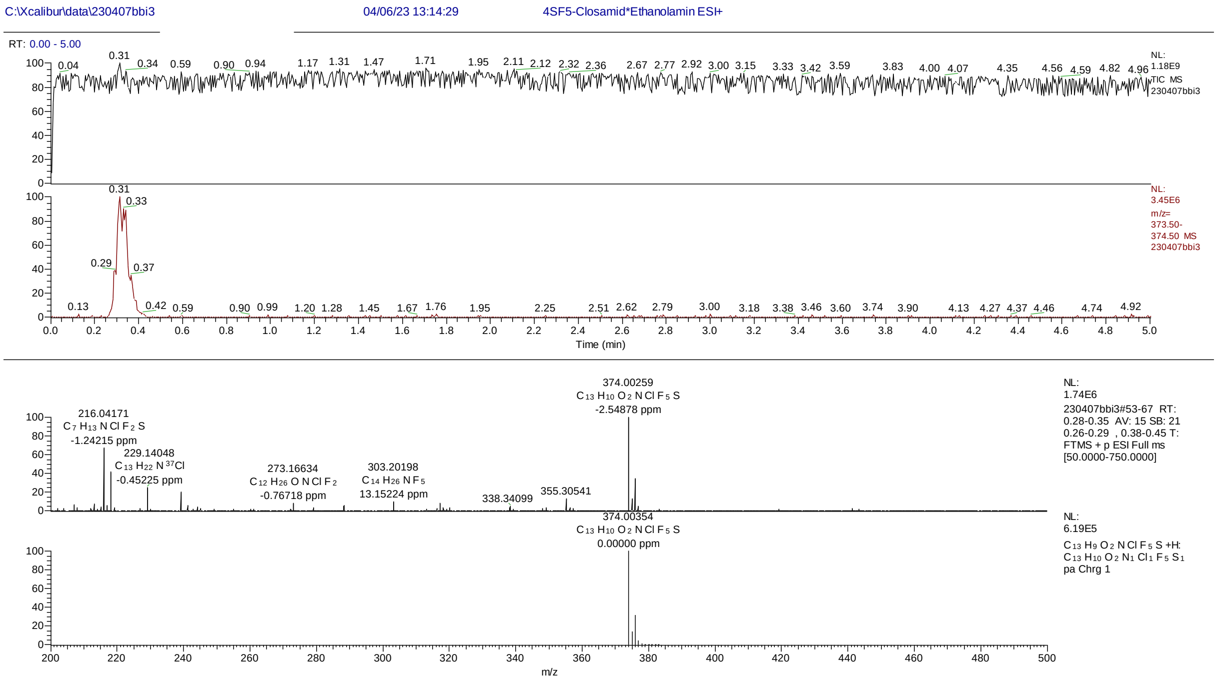

Figure S16. ESI-HRMS (ESI+) spectrum of **3a**.

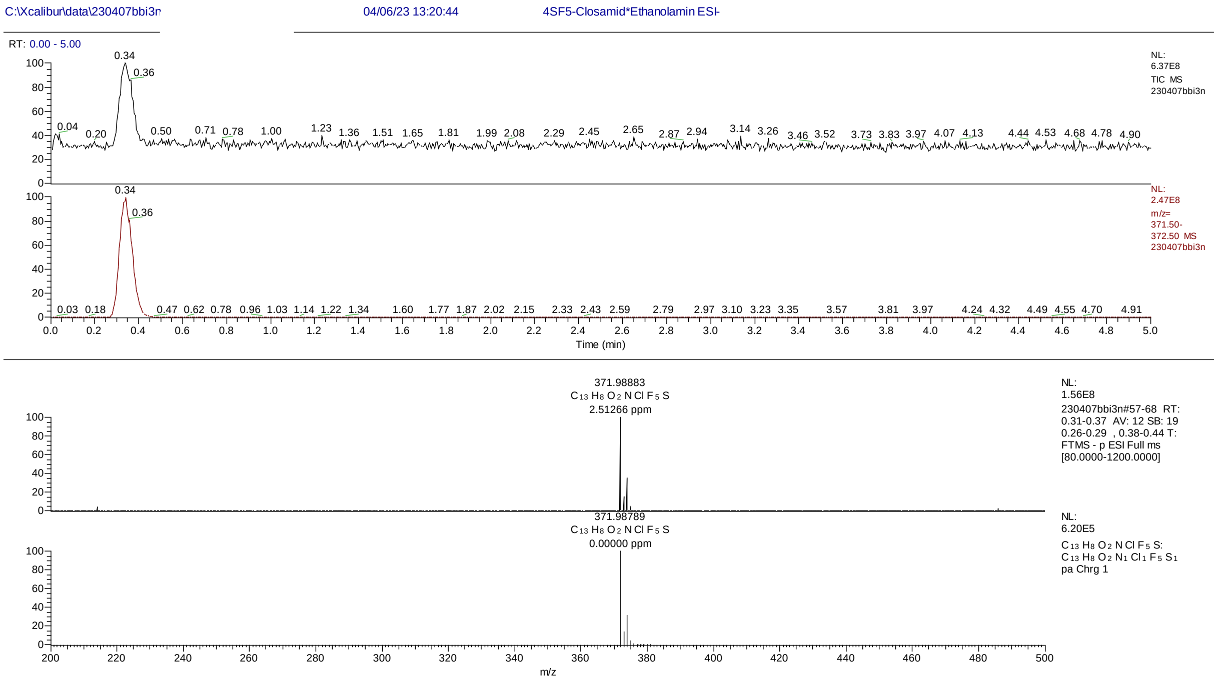

Figure S17. ESI-HRMS (ESI-) spectrum of **3a**.

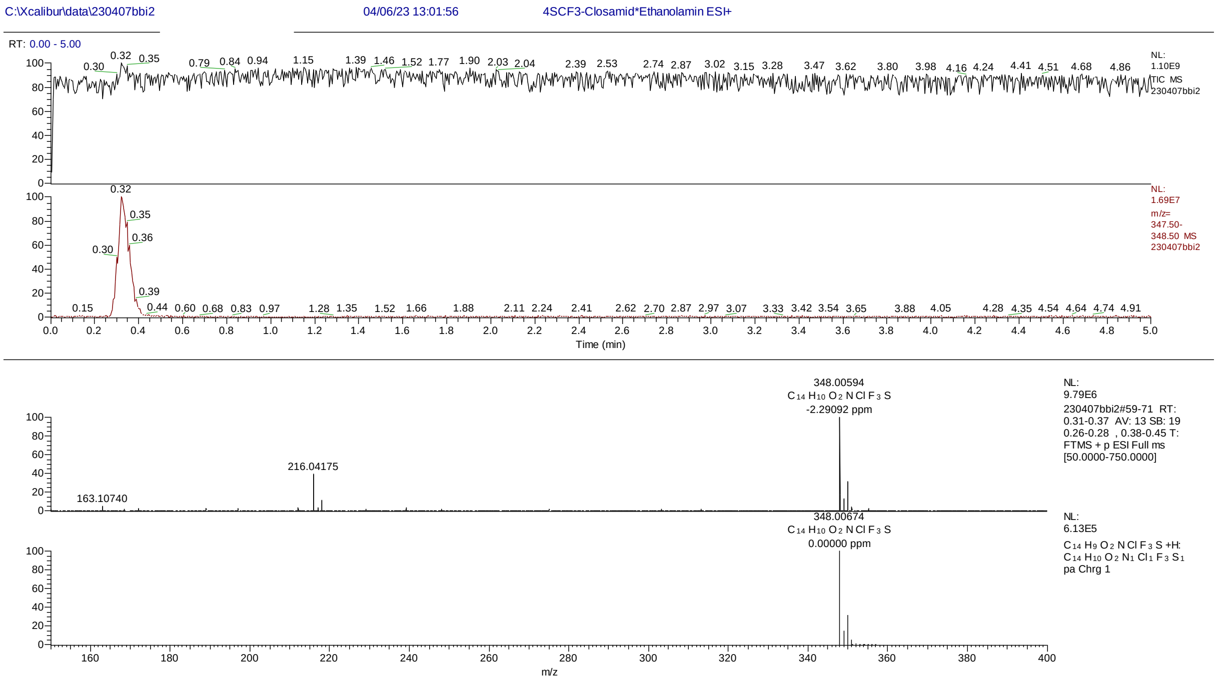

Figure S18. ESI-HRMS (ESI+) spectrum of **3b**.

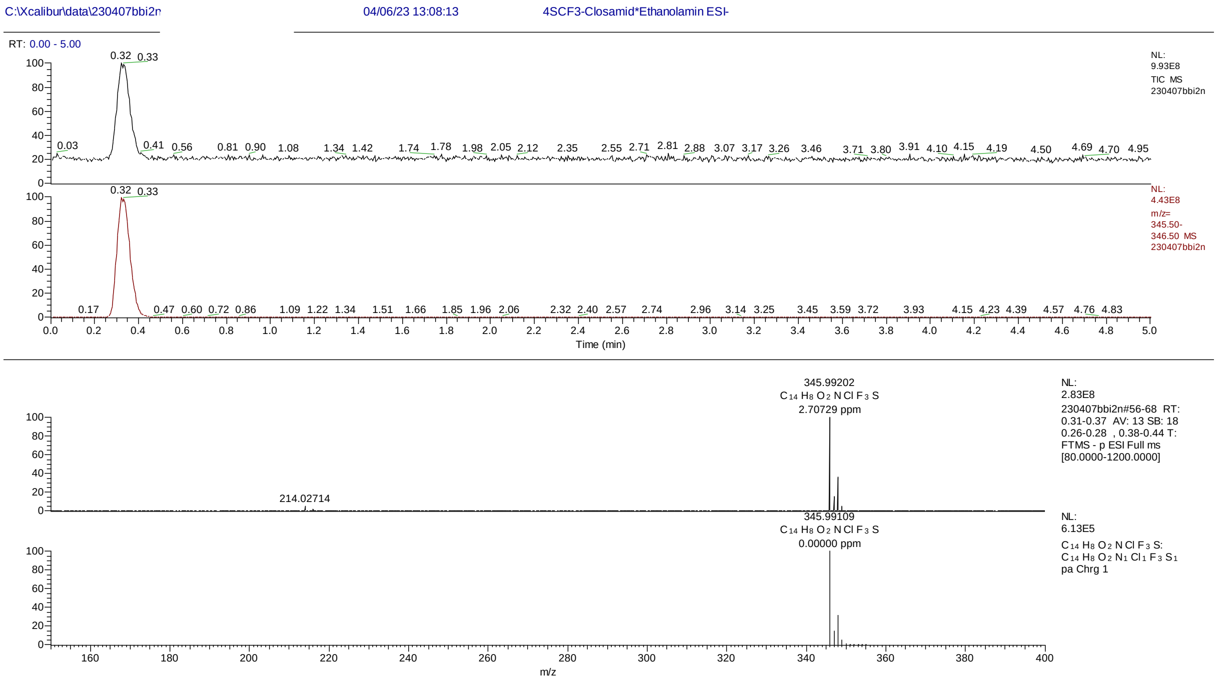

Figure S19. ESI-HRMS (ESI-) spectrum of **3b**.
